# Integrated Collaborative Care for Youths With Mental Health and Substance Use Challenges: A Randomized Clinical Trial

**DOI:** 10.1001/jamanetworkopen.2025.9565

**Published:** 2025-05-13

**Authors:** Jo Henderson, Peter Szatmari, Kristin Cleverley, Clement Ma, Lisa D. Hawke, Amy Cheung, Jacqueline Relihan, Mahalia Dixon, Meaghen Quinlan-Davidson, Myla Moretti, Claire de Oliveira, Alina Lee, Darren B. Courtney, David O’Brien, Heather McDonald, Krista Lemke, Tony Pignatiello, Suneeta Monga, Nicole Kozloff, Leigh Solomon, Brendan F. Andrade, Melanie Barwick, Alice Charach, Lynn Courey, Karleigh Darnay, Paul Kurdyak, Elizabeth Lin, Di Shan

**Affiliations:** 1 Margaret and Wallace McCain Centre for Child, Youth and Family Mental Health, Centre for Addiction and Mental Health, Toronto, Ontario, Canada; 2 Department of Psychiatry, University of Toronto, Toronto, Ontario, Canada; 3 Cundill Centre for Child and Youth Depression, Centre for Addiction and Mental Health, Toronto, Ontario, Canada; 4 Department of Psychiatry, Hospital for Sick Children, Toronto, Ontario, Canada; 5 Lawrence S. Bloomberg Faculty of Nursing and Faculty of Medicine, Centre for Addiction and Mental Health, Toronto, Ontario, Canada; 6 Biostatistics Core Services, Centre for Addiction and Mental Health, Toronto, Ontario, Canada; 7 Division of Biostatistics, Dalla Lana School of Public Health, University of Toronto, Toronto, Ontario, Canada; 8 Education Department, Collaborative Learning College, Centre for Addiction and Mental Health, Toronto, Ontario, Canada; 9 Youth and Family Mental Health, Margaret and Wallace McCain Centre for Child Youth and Family Mental Health, Centre for Addiction and Mental Health, Toronto, Ontario, Canada; 10 Dalla Lana School of Public Health, University of Toronto, Toronto, Ontario, Canada; 11 Institute of Health Policy, Management and Evaluation, University of Toronto, Toronto, Ontario, Canada; 12 Child Health Evaluative Sciences, The Hospital for Sick Children Research Institute, Toronto, Ontario, Canada; 13 Institute for Mental Health Policy Research, Centre for Addiction and Mental Health, Toronto, Ontario, Canada; 14 Campbell Family Mental Health Research Institute, Centre for Addiction and Mental Health, Toronto, Ontario, Canada; 15 Institute for Clinical Evaluative Sciences, Toronto, Ontario, Canada; 16 Biostatistics Core, Center for Complex Interventions, Centre for Addiction and Mental Health, Toronto, Ontario, Canada; 17 Yorktown Family Services, Toronto, Ontario, Canada; 18 LOFT Community Services, Toronto, Ontario, Canada; 19 Michael Garron Hospital, Toronto East Health Network, Toronto, Ontario, Canada; 20 Temerty Faculty of Medicine, Hospital for Sick Children, University of Toronto, Toronto, Ontario, Canada; 21 Slaight Family Centre for Youth in Transition, Centre for Addiction and Mental Health, Toronto, Ontario, Canada; 22 Department of Psychiatry, North York General Hospital, University of Toronto, Toronto, Ontario, Canada; 23 Department of Psychiatry, Dalla Lana School of Public Health, Hospital for Sick Children Research Institute, University of Toronto, Toronto, Ontario, Canada; 24 Division of Child and Youth Psychiatry, Department of Psychiatry, University of Toronto, Hospital for Sick Children Research Institute, Toronto, Ontario, Canada; 25 The Sashbear Foundation, Toronto, Ontario, Canada; 26 Youth Wellness Hubs Ontario, Centre for Addiction and Mental Health, Toronto, Ontario, Canada; 27 Canadian Institute for Health Information, Toronto, Ontario, Canada

## Abstract

**Importance:**

Research on the integrated collaborative care team (ICCT) model, a version of an integrated youth service, with youths and families is needed to evaluate its effectiveness in improving mental health functioning compared with hospital outpatient treatment as usual (TAU).

**Objectives:**

To test the benefits of the ICCT in improving youth functioning compared with TAU, to assess youths’ general psychopathology symptoms and substance use problems, and to quantify health service access, use, and satisfaction.

**Design, Setting, and Participants:**

This 2-group pragmatic randomized clinical trial enrolled youths (14-17 years) and caregivers in Canada from September 2016 to March 2020. Participants were randomized to either 1 of 5 outpatient mental health hospital programs or 1 of 3 community ICCTs. Data analyses began on July 12, 2021, and concluded on November 12, 2023.

**Intervention:**

Youths were offered services in the ICCT or TAU groups. Outcomes were assessed at baseline, 6 months, and 12 months.

**Main Outcomes and Measures:**

The primary outcome was change in youth-reported mental health functioning as measured with the Columbia Impairment Scale (CIS). Secondary outcomes included the following: (1) caregiver-reported functioning and youth- and caregiver-reported general psychopathology and substance use, (2) mental health service satisfaction, and (3) health service access and use. Linear mixed-effects and generalized estimating equation models were used to compare outcomes in intention-to-treat analyses.

**Results:**

This study included 247 youths; 124 were randomized to the ICCT and 123 were randomized to TAU. There were no baseline differences between groups; youths had a mean (SD) age of 15.7 (1.1) years. A total of 85 (34.4%) youths identified as boys or men, 157 (63.6%) identified as girls or women, and 5 (2.0%) identified as transgender, reported diverse gender identities, or were missing these data. CIS scores improved over the 12 months for both the ICCT group (Cohen *d* = −3.59 [95% CI, −4.99 to −2.20]; *P* < .001) and the TAU group (Cohen *d* = −2.59 [95% CI, −4.01 to −1.18]; *P* < .001). Significant differences in changes between groups were not observed (unadjusted CIS model, partial η^2^ = 0.002; *P* = .59). Both groups had mean scores suggesting satisfaction with services. The ICCT group accessed services sooner (median, 9 days; IQR, 5-16 days) compared with the TAU group (median, 27 days; IQR, 14-57 days) (Cohen *d* = 0.54 [95% CI, 0.27-0.81]; *P* < .001, *t* test). Fewer youths in the ICCT group saw a psychiatrist compared with youths in the TAU group (22 [17.5%] vs 104 [82.5%]; *P* < .001, χ^2^ test; φ = −0.67).

**Conclusions and Relevance:**

Although no clinical differences between groups were observed in this trial, youths receiving ICCT care improved in multiple metrics, accessed services sooner, and used fewer psychiatric resources than those in TAU programs. Future research should focus on how ICCT models can integrate and collaborate with hospital outpatient services.

**Trial Registration:**

ClinicalTrials.gov Identifier: NCT02836080

## Key Points

**Question:**

What is the effectiveness of an integrated collaborative care team (ICCT) model of integrated youth services compared with hospital-based outpatient mental health services (treatment as usual [TAU]) in improving mental health functioning among youths aged 14 to 17 years?

**Findings:**

In this randomized clinical trial involving 247 youths, no differences in change in functioning or mental health and substance outcomes between the ICCT and TAU treatment groups were observed. Youths in both groups experienced significant improvement in functioning and mental health and substance use outcomes.

**Meaning:**

These results suggest that the ICCT model is associated with improved youth functioning and mental health and substance use outcomes that are no different from outpatient hospital care.

## Teaser

This randomized clinical trial examines the effectiveness of an integrated collaborative care team model of integrated services for youths aged 14 to 17 years with mental health and substance use challenges compared with usual care over 12 months.

## Introduction

Approximately 20% of youths are affected by mental health and substance use (MHSU) disorders.^1^ Most youths with MHSU concerns do not receive evidence-based treatments^1^ and face barriers to care.^2,3^ These barriers exist despite the personal, social, and economic costs of failing to provide adequate MHSU interventions.^4^ Mental health difficulties account for a substantial proportion of the overall burden of disease among youths aged 10 to 24 years globally.^5^

Integrated models of service delivery for youths (known as integrated youth services [IYSs]), such as headspace in Australia^3^ and Jigsaw in Ireland,^6^ have emerged as innovative models of MHSU services for youths.^7,8^ Codesigned with youths, families, health care practitioners, and those with system expertise, these integrated models are multidisciplinary and bring together intersectoral services^7,8^ tailored to individuals through needs-based planning and measurement-based care (MBC), delivered in youth-friendly settings.^7,8^ Services usually include evidence-based psychotherapies, primary care, peer and family supports, and social services.^7,8^

Despite their promise and implementation globally,^7,8^ we are unaware of any controlled clinical trial evaluating these integrated models of youth services. Evidence supporting these models has been based on quasi-experimental or pre-post program evaluations,^7,9,10^ which insufficiently guide policymakers and clinicians.

We developed a variation of headspace,^3,11,12^ the integrated collaborative care team (ICCT) model, which allocates several coordinated community agencies’ existing psychosocial resources based on urgency of youth needs. We collaboratively designed the ICCT model based on the literature^7^ (eFigure 1 in Supplement 1). Youths and families were engaged at each step to increase ICCT relevance and impact^13,14,15^ and facilitate its uptake and spread (eTable 1 in Supplement 1).

We evaluated the ICCT model as part of a pragmatic randomized clinical trial (RCT), comparing ICCT with usual care in outpatient youth mental health hospital services. This study aimed to answer the following question: Among youths aged 14 to 17 years with MHSU problems, is ICCT more effective in reducing functional impairment compared with hospital-based mental health outpatient treatment as usual (TAU) over a 12-month period?

## Methods

### Trial Design

For this pragmatic RCT, we engaged youths and families in project leadership, governance, study design, implementation, and interpretation. Information on their engagement has been published elsewhere^15,16^ and is summarized in eTable 1 in Supplement 1.^17^ The McCain model of youth engagement^18^ informed codesign approaches with youths and family members.^15,16^ This 2-group superiority pragmatic RCT had 1:1 allocation to the ICCT or TAU. Randomization was at the level of the individual participant. The pragmatic study tested the intervention under everyday services.^19^ Research ethics board approval was obtained by the hospital sites. The trial protocol was published previously^3^ (Supplement 2). Informed consent was obtained from youths and caregivers at the intake visit prior to enrollment and randomization. There were no deviations from the published protocol.^3^ The study followed the Consolidated Standards of Reporting Trials (CONSORT) guideline. We used the Pragmatic Explanatory Continuum Indicator Summary (PRECIS) guideline to retrospectively assess pragmatism (eTable 2 in Supplement 1).

### Setting and Enrollment

Hospital sites included 1 psychiatric hospital (Centre for Addiction and Mental Health), 3 general hospitals (Sunnybrook Hospital, Michael Garron Hospital, and North York General Hospital), and 1 children’s hospital (The Hospital for Sick Children) in Toronto, Canada. ICCT sites included Central Toronto (Skylark Children, Youth and Families), Toronto East (Danforth), and Scarborough (East Metro Youth Services) in Toronto. Eligible youths were recruited at hospital intake and randomly allocated to either TAU at that hospital’s youth psychiatric outpatient services or to the participant’s choice of 1 of 3 ICCTs. Participants were enrolled from September 2016 to March 2020; enrollment was terminated in March 2020 due to COVID-19 restrictions.^20^

### Participants

Eligibility criteria included being aged 14 to 17 years at intake referred for MHSU problems and being eligible to receive any outpatient mental health service at the participating hospitals. Referrals to specialty clinics (eg, autism, eating disorders, urgent care) were excluded. Primary caregiver participation was encouraged although optional. The Figure and the eMethods in Supplement 1 present participant exclusion criteria, identification, referral, and randomization details. Youths and caregivers received an honorarium at each measurement time point.

**Figure.**
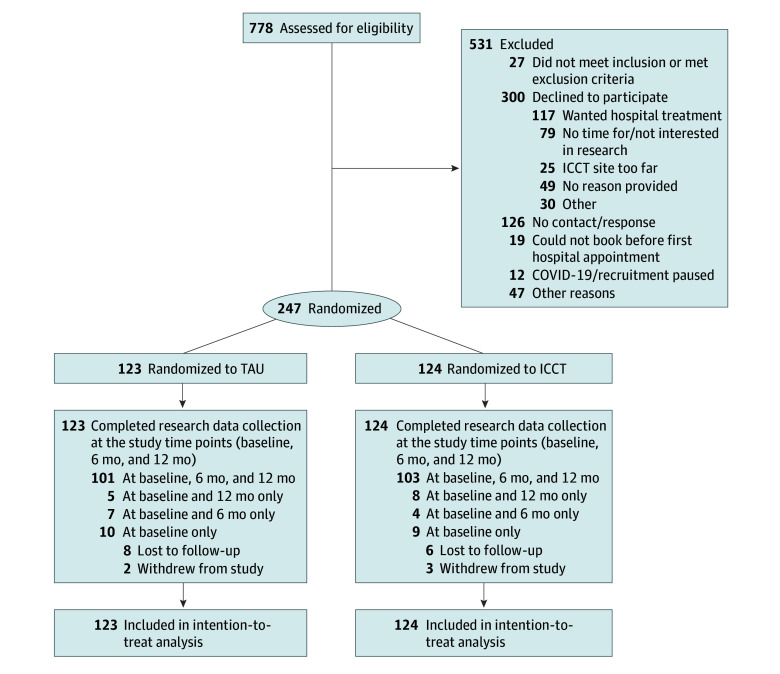
Study Flow Diagram ICCT indicates integrated collaborative care team; TAU, treatment as usual.

### Randomization and Blinding

Research assistants used a randomized block design to allocate participants into TAU or ICCT groups. Random block sizes were used within 2 strata defined by sex assigned at birth (male or female) at each hospital (eMethods in Supplement 1). REDCap (Research Electronic Data Capture) software^21^ was used for randomization. The allocation sequence was concealed to the research assistant until after baseline data collection and randomization. All other team members, including independent outcome assessors, the research team, and biostatisticians, were blinded to the treatment allocation. Clinicians, youths, and caregivers were not blinded to treatment allocation.

#### Integrated Collaborative Care Teams

ICCT services were provided at 3 community sites across the greater Toronto area. Participants received treatment ranging in intensity based on need from a multidisciplinary clinical team (eg, psychiatrist, social worker, and nurse practitioner), trained peer support workers, primary health care practitioners, and care navigators supporting care coordination (more details available in Henderson et al^3,14^). On-site staff were trained on the model and fidelity measures by the research team and partnering agencies.^14^ All youths were initially provided a single solution-focused brief therapy (SFBT) session to identify needs and provide problem-solving support. Youths requiring more than SFBT were allocated to the moderate-intensity service, which involved group dialectical behavior therapy (DBT). Youths showing signs of potential psychosis or with plans for self-harm required higher-intensity services and were immediately referred to a consultation with an ICCT child and youth psychiatrist. If there was no response to DBT treatment according to an MBC protocol or the patient requested medication, a consultation with a psychiatrist was also provided. Services switched from in-person to virtual in March 2020 following COVID-19 restrictions.^20^ Services received were tracked through medical record review (details available in Henderson et al^3,14^).

#### Treatment as Usual

Participants randomized to TAU received standard outpatient services at 1 of the 5 hospitals. TAU services were tracked through medical record review (details available in Henderson et al^3,14^). TAU most often comprised an initial assessment by a psychiatrist or social worker, a subsequent referral to group or individual cognitive behavior therapy, or other services as needed.

### Measures and Instruments

Baseline characteristics assessed included sex, gender, and psychiatric diagnosis. Sex assigned at birth was collected at intake. Self-report data on gender identity (coded as boy/man, girl/woman, or transgender and diverse gender identities, such as Two-Spirit, genderqueer, or androgynous) were also collected. See the eMethods in Supplement 1 for sociodemographics.

Data on ancestry or ethnic group or cultural background were also collected because we wanted to identify and understand health inequities. These data were collected through open-ended responses and were provided by patients as follows: Black, East Asian, Indigenous (First Nations and Métis), Latin American, Middle Eastern, mixed heritage, West Indian, South Asian, White, other race or ethnicity (responses included Canadian Jewish; Guyanese, St Lucian; mixed Latin American and White North American; French Canadian, Italian, Métis, Scottish, South African; Greek; and Spanish White and South East Asian), or unknown.

Trained research staff evaluated youths for psychiatric diagnoses (meeting *Diagnostic and Statistical Manual of Mental Disorders* (Fifth Edition) [*DSM-5*] criteria) using the semistructured Diagnostic Interview for Affective and Anxiety Disorders–Child Version (DIAS-C),^22^ which is an updated version of the Kiddie Schedule for Affective Disorders and Schizophrenia.^23^ A *DSM-5* disorder was operationalized as having a definite^24^ diagnosis by either the youth or the caregiver.

### Outcomes

#### Primary Outcome

Youth representatives selected the Columbia Impairment Scale (CIS; youth self-report version^25^) as the primary outcome to assess mental health functioning. The 13-item scale measures interpersonal relations, broad psychopathology, and school, work, and leisure functioning using a 5-point scale ranging from 0 (no problem) to 4 (very bad problem) over the prior 2 weeks (score range, 0-52). A sum score is used, in which higher scores indicate more functional impairment.^3^ The CIS was administered at baseline, 6 months, and 12 months through REDCap.

#### Secondary Outcomes

Secondary outcomes included the following: (1) caregiver-reported functioning and youth- and caregiver-reported general psychopathology and substance use, (2) mental health service satisfaction, and (3) health service access and use. The eMethods in Supplement 1 provide a description of the secondary outcome measures.

Caregiver-reported youth impairment on the CIS was a secondary outcome.^3,25^ Youths and caregivers also completed the Strengths and Difficulties Questionnaire (SDQ)^26^ to measure general psychopathology over the prior 6 months. A total SDQ score was calculated, excluding the prosocial subscale (score range, 0-40). Youths completed the Substance Use Problems Subscale of the Global Appraisal of Individual Needs–Short Screener (GAIN-SS), version 3.^27^ GAIN-SS scores were dichotomized as (1) no substance use problems in the last 3 months or (2) any substance use problems in the last 3 months (score range, 0-5). Both the SDQ and GAIN-SS were completed at baseline, 6 months, and 12 months.

To measure service satisfaction at 6 and 12 months (score range, 1-4), youths and caregivers completed the client and family or supporters’ versions of the Ontario Perception of Care Tool for Mental Health and Addictions (OPOC-MHA), respectively.^28^ Higher scores indicated more positive service experience.

Finally, data on health service access and use were collected in the medical record review. They were operationalized as (1) wait time to the first clinical intervention and (2) number of participants who had a consultation with a psychiatrist at any point from baseline to 12 months.

### Statistical Analysis

We performed statistical analyses using SAS Enterprise Guide, version 7.1 (SAS Institute Inc).^29,30^ We used mean imputation to impute missing items (or items rated as “not applicable”) from the outcome scales. If a measure was missing more than 50% of items, we omitted the observation from the analysis. “Not applicable” responses (coded as a response of 5) were only present for the primary outcome, CIS score (eTable 3 in Supplement 1).

We also conducted intention-to-treat analyses. Descriptive statistics were used to summarize self-reported youth and caregiver baseline characteristics by treatment group and overall. We used linear mixed-effects models (LMMs) with random intercepts and an unstructured covariance matrix to model associations between the treatment groups, time (categorical), and treatment-by-time interactions on CIS and SDQ scores across 3 time points (baseline, 6 months, and 12 months) for both youths and caregivers, adjusting for baseline scores (unadjusted models). Generalized estimating equations (GEEs) were used to analyze associations of treatment group, time (as a categorical measure), and treatment group-by-time interactions on the binary GAIN-SS scores across baseline, 6 months, and 12 months for youth participants. Standardized partial η^2^ effects were computed for the treatment-by-time interaction using the SAS macro.^31^ Two-sample *t* tests were used for between-group comparisons of OPOC-MHA scores at 6 and 12 months for youths and caregivers. Pairwise comparisons of functioning and MHSU problems were estimated per time point and treatment group.

Estimated marginal means, odds ratios (ORs), and respective 95% CIs are reported; estimated means and probabilities for adjusted models assume the average value of model covariates. Health service access and use between groups were compared using 2-sample *t* tests and χ^2^ tests. Standardized effect sizes, Cohen *d* values, and φ coefficients^32^ were computed for 2-sample *t* tests and χ^2^ tests. The original estimated sample size of 500 participants would have achieved 80% power to detect a small to medium between-group difference in CIS scores of 0.15 SD at 6 months and 0.30 SD at 12 months, assuming an intraclass correlation coefficient of 0.4 or less between 6 and 12 months.^3^ We were unable to reach our desired sample size due to the COVID-19 pandemic. The actual sample size of 247 participants only achieved 49.8% power to detect the planned effect size. Sensitivity analyses were performed for LMM and GEE models for youth participants, adjusting for gender identity, age at baseline, and enrolling study site (adjusted models). The eMethods in Supplement 1 provides details regarding sensitivity analyses, significance level, and secondary outcome analysis. Data analyses began on July 12, 2021, and concluded on November 12, 2023.

## Results

### Sample Description

Of the 778 youths assessed, 247 (31.7%) met the eligibility criteria, wished to participate, and were randomized to the ICCT (n = 124) or TAU (n = 123) (Figure). Demographic characteristics were balanced across groups (Table 1). Youths had a mean (SD) age of 15.7 (1.1) years. A total of 85 (34.4%) youths identified as boys or men, 157 (63.6%) identified as girls or women, and 5 (2.0%) identified as transgender, reported diverse gender identities, or were missing these data. Youths reported their ancestry or ethnic group or cultural background as Black (10 [4.1%]), East Asian (17 [6.9%]), Indigenous (First Nations and Métis) (2 [0.8%]), Latin American (19 [7.7%]), Middle Eastern (5 [2.0%]), mixed heritage (22 [8.9%]), West Indian (6 [2.4%]), South Asian (15 [6.1%]), White (137 [55.5%]), or other race or ethnicity (7 [2.8%]); these data were unknown for 7 youths (2.8%).

**Table 1.** Demographic Characteristics of Youth and Caregiver Participants at Baseline^a^

Characteristic	Overall	ICCT group	TAU group
**Youths**			
No. of youths	247	124	123
Age at enrollment, y			
14	43 (17.5)	19 (15.3)	24 (19.7)
15	65 (26.4)	34 (27.4)	31 (25.4)
16	70 (28.5)	34 (27.4)	36 (29.5)
17	68 (27.6)	37 (29.8)	31 (25.4)
Missing	1	0	1
Gender identity			
Boy or man	85 (34.6)	42 (34.1)	43 (35.0)
Girl or woman	157 (63.8)	79 (64.2)	78 (63.4)
Transgender, diverse gender identities, or missing	5 (2.0)	3 (2.4)	2 (1.6)
Student status			
Yes, full-time	236 (95.6)	118 (95.2)	118 (95.9)
Yes, part-time	6 (2.4)	2 (1.6)	4 (3.3)
No	5 (2.0)	4 (3.2)	1 (0.8)
Born in Canada			
No	35 (14.2)	23 (18.6)	12 (9.8)
Yes	212 (85.8)	101 (81.5)	111 (90.2)
Ancestry or ethnic group or cultural background			
Black	10 (4.1)	6 (4.8)	4 (3.3)
East Asian	17 (6.9)	9 (7.3)	8 (6.5)
Indigenous (First Nations and Métis)	2 (0.8)	1 (0.8)	1 (0.8)
Latin American	19 (7.7)	9 (7.3)	10 (8.1)
Middle Eastern	5 (2.0)	1 (0.8)	4 (3.3)
Mixed heritage	22 (8.9)	8 (6.5)	14 (11.4)
West Indian	6 (2.4)	4 (3.2)	2 (1.6)
South Asian	15 (6.1)	6 (4.8)	9 (7.3)
White	137 (55.5)	73 (58.9)	64 (52.0)
Other race or ethnicity^b^	7 (2.8)	3 (2.4)	4 (3.3)
Unknown	7 (2.8)	4 (3.2)	3 (2.4)
**Caregivers**			
No. of caregivers	189	95	94
Age at enrollment, median (range) [No.], y	48 (20-83) [183]	47 (30-80) [92]	48 (20-830) [91]
Gender identity			
Boy or man	19 (10.1)	8 (8.4)	11 (11.8)
Girl or woman	169 (89.9)	87 (91.6)	82 (88.2)
Transgender, diverse gender identities, or missing	1 (0.5)	0	1 (1.1)
Income, $			
0-29 999	29 (15.8)	13 (14.1)	16 (17.4)
30 000-59 999	29 (15.8)	16 (17.4)	13 (14.1)
60 000-89 999	13 (7.1)	4 (4.4)	9 (9.8)
90 000-119 999	26 (14.1)	14 (15.2)	12 (13.0)
120 000-149 999	25 (13.6)	12 (13.0)	13 (14.1)
≥150 000	62 (33.7)	33 (35.9)	29 (31.5)
Missing	5	3	2

Abbreviations: ICCT, integrated collaborative care team; TAU, treatment as usual.

^a^ Unless otherwise indicated, values are presented as No. (%) of participants.

^b^ These data were collected through open-ended responses and were provided as follows: Canadian Jewish; Guyanese, St Lucian; mixed Latin American and White North American; French Canadian, Italian, Métis, Scottish, South African; Greek; and Spanish White and South East Asian.

eTable 4 in Supplement 1 presents demographics by complete and incomplete CIS scores at baseline. A total of 189 youths (76.5%) had caregivers who participated at baseline (Table 1). Measure completion was 82.0% or greater at each follow-up assessment and similar across groups. eTable 5 in Supplement 1 presents the results of DIAS-C interviews. Attrition rates were 16.9% (21 of 124) for the ICCT group and 17.9% (22 of 123) for the TAU group (Figure).

### Outcomes

#### Youth-Reported Mental Health Functioning

Pairwise contrasts within groups showed that youths in the ICCT group had significantly lower mean CIS scores at 12 months, indicating improved mental health functioning compared with baseline (*d* = −3.59 [95% CI, −4.99 to −2.20]; *P* < .001). Similarly, youths in the TAU group had significantly lower mean CIS scores at 12 months compared with baseline (*d* = −2.59 [95% CI, −4.01 to −1.18]; *P* < .001) (Table 2 and eFigure 2 in Supplement 1). These differences remained even after controlling for covariates (eFigure 3 in Supplement 1). However, there were no significant treatment-by-time interactions or differences in change in youth CIS scores in the ICCT group compared with the TAU group across the 3 time points in the unadjusted model (partial η^2^ = 0.002; *P* = .59 for treatment group-by-time interaction) (Table 2) or the adjusted model (η^2^ = 0.003; *P* = .56 for interaction). eTable 6 in Supplement 1 presents the complete case analysis.

**Table 2.** Mental Health Functioning and General Psychopathology Among Youths Across 3 Time Points^a^

Outcome	Estimated marginal means (95% CI)			*P* value, treatment-by-time interaction	Pairwise contrasts within groups, difference (95% CI)^b^	Pairwise contrasts between ICCT and TAU groups (95% CI)		
	Baseline	6 mo	12 mo			Baseline	6 mo	12 mo
**Columbia Impairment Scale**								
Youth-reported score								
Unadjusted (n = 247)								
ICCT	21.44 (20.36-22.52)	18.23 (17.07-19.39)	17.85 (16.71-8.99)	.59	−3.59 (−4.99 to −2.20)	0.28 (−1.25 to 1.82)	−0.44 (−2.08 to 1.20)	−0.72 (−2.35 to 0.91)
TAU	21.16 (20.07-22.24)	18.66 (17.51-19.82)	18.56 (17.40-19.73)		−2.59 (−4.01 to −1.18)			
Adjusted by age, site, and gender identity (n = 246)								
ICCT	20.88 (19.12-22.64)	17.63 (15.82-19.43)	17.28 (15.49-19.07)	.56	−3.60 (−5.00 to −2.20)	0.35 (−1.20 to 1.89)	−0.38 (−2.02 to 1.27)	−0.73 (−2.37 to 0.91)
TAU	20.53 (18.75-22.32)	18.00 (16.18-19.83)	18.00 (16.16-19.85)		−2.53 (−3.95 to −1.11)			
Caregiver-reported youth scores								
Unadjusted (n = 189)								
ICCT	21.13 (19.99-22.27)	17.92 (17.20-19.70)	18.45 (16.65-19.18)	.75	−3.21 (−4.77 to −1.65)	−0.14 (−1.75 to 1.48)	−0.97 (−2.75 to 0.81)	−0.59 (−2.40 to 1.22)
TAU	21.27 (−22.41 to 20.12)	19.42 (18.16-20.69)	18.50 (17.21-19.79)		−2.76 (−4.35 to −1.18)			
Adjusted by age, site, and gender identity (n = 188)								
ICCT	21.60 (19.91-23.29)	18.89 (17.14-20.65)	18.39 (16.63-10.15)	.74	−3.21 (−4.77 to −1.64)	−0.14 (−1.76 to 1.48)	−1.01 (−2.80 to 0.77)	−0.58 (−2.39 to 1.24)
TAU	21.74 (20.02-23.46)	19.91 (18.11-21.70)	18.97 (17.13-20.82)		−2.77 (−4.36 to −1.18)			
**Strengths and Difficulties Questionnaire**								
Youth-reported score								
Unadjusted (n = 247)								
ICCT	20.15 (19.61-20.70)	18.90 (18.31-19.49)	18.83 (18.25-19.41)	.14	−1.32 (−2.05 to −0.60)	0.13 (−0.64 to 0.91)	0.94 (0.11-1.76)	1.09 (0.26-1.91)
TAU	20.02 (19.47-20.57)	17.96 (17.38-18.55)	17.74 (17.16-18.33)		−2.28 (−3.01 to −1.55)			
Adjusted by age, site, and gender identity (n = 246)								
ICCT	19.71 (18.84-20.59)	18.46 (17.56-19.35)	18.39 (17.50-19.28)	.12	−1.33 (−2.05 to −0.60)	0.14 (−0.64 to 0.92)	0.96 (0.13-1.79)	1.15 (0.32-1.98)
TAU	19.57 (18.68-20.46)	17.50 (16.59-18.41)	17.24 (16.33-18.16)		−2.33 (−3.06 to −2.00)			
Caregiver-reported youth scores								
Unadjusted (n = 189)								
ICCT	18.73 (18.13-19.34)	17.02 (16.36-17.69)	16.99 (16.32-17.67)	.84	−1.74 (−2.58 to −0.90)	0.16 (−0.70 to 1.02)	−0.14 (−1.08 to 0.81)	0.20 (−0.76 to 1.16)
TAU	18.58 (17.97-19.18)	17.16 (16.49-17.83)	16.79 (16.10-17.48)		−1.78 (−2.63 to −0.93)			
Adjusted by age, site, and gender identity (n = 188)								
ICCT	18.78 (17.89-19.68)	17.06 (16.13-17.99)	17.06 (16.13-17.99)	.86	−1.72 (−2.57 to −0.88)	0.16 (−0.70 to 1.02)	−0.12 (−1.07 to 0.84)	0.21 (−0.76 to 1.18)
TAU	18.62 (17.71-19.54)	17.18 (16.22-18.13)	16.85 (15.87-17.83)		−1.77 (−2.63 to −0.92)			

Abbreviations: ICCT, integrated collaborative care team; TAU, treatment as usual.

^a^ Estimated marginal means for adjusted models assume the average value of model covariates.

^b^ Differences between baseline and 12 months.

#### Caregiver-Reported Functioning and Caregiver- and Youth-Reported General Psychopathology and Substance Use, Mental Health Service Satisfaction, and Health Service Access and Use

Caregiver-reported CIS scores, youth- and caregiver-reported SDQ scores, and youth- and caregiver-reported OPOC-MHA scores are presented in eTable 6 and in the eResults in Supplement 1. For each variable, there were significant changes between 12 months and baseline, but there were no significant differences between groups.

GAIN-SS scores suggested that youths in the ICCT and TAU groups may have had a positive substance use disorder screening result across the 3 time points in the unadjusted model (OR, 0.80 [95% CI, 0.57-1.14] vs 1.28 [95% CI, 0.82-1.99]; *P* = .07 for treatment group-by-time interaction) and in the adjusted model (OR, 1.40 [95% CI, 0.96-0.25] vs 0.79 [95% CI, 0.53-1.18]; *P* = .10). Pairwise contrasts did not indicate significant differences in substance use disorder between time and treatment group (Table 3 and eFigure 2 in Supplement 1). At baseline, youths in the ICCT group appeared more likely to have problematic substance use than youths in the TAU group (predicted probability, 0.35 [95% CI, 0.28-0.44] vs 0.29 [95% CI, 0.22-0.38]), but the difference was not statistically significant. However, in both the ICCT and TAU groups, this effect was reversed at 6 months (predicted probability 0.28 [95% CI, 0.20-0.37] vs 0.34 [0.26-0.44]) and 12 months (predicted probability, 0.31 [95% CI, 0.23-0.40] vs 0.35 [95% CI, 0.26-0.44]).

**Table 3.** Estimated Odds of Positive Substance Use Disorder Screening Among Youths Between and Within Treatment Groups Across 3 Time Points^a^

Youth-reported score (N = 247)	Predicted probability (95% CI)			*P* value, treatment-by-time interaction	Pairwise contrasts within groups, OR (95% CI)^b^	Pairwise contrasts between ICCT vs TAU groups, OR (95% CI)		
	Baseline	6 mo	12 mo			Baseline	6 mo	12 mo
Unadjusted								
ICCT	0.35 (0.28-0.44)	0.28 (0.20-0.37)	0.31 (0.23-0.40)	.07	0.80 (0.57-1.14)	1.33 (0.78-2.27)	0.75 (0.42-1.34)	0.82 (0.47-1.47)
TAU	0.29 (0.22-0.38)	0.34 (0.26-0.44)	0.35 (0.26-0.44)		1.28 (0.82-1.99)			
Adjusted by age, site, and gender identity								
ICCT	0.30 (0.16-0.50)	0.24 (0.11-0.43)	0.26 (0.13-0.45)	.10	1.40 (0.96-0.25)	1.33 (0.77-2.32)	0.76 (0.41-1.39)	0.81 (0.45-1.47)
TAU	0.25 (0.11-0.46)	0.29 (0.14-0.52)	0.30 (0.15-0.51)		0.79 (0.53-1.18)			

Abbreviations: ICCT, integrated collaborative care team; OR, odds ratio; TAU, treatment as usual.

^a^ Screens were completed using the Global Appraisal of Individual Needs–Short Screener. Predicted probabilities for adjusted models assume the average value of model covariates.

^b^ Differences between baseline and 12 months.

With regard to mental health service access and use, median wait time from randomization to first clinical intervention visit was shorter in the ICCT group (9 days; IQR, 5-16 days) compared with the TAU group (27 days; IQR, 14-57 days) (Cohen *d* = 0.54 [95% CI, 0.27-0.81]; *P* < .001, *t* test). A total of 22 youths (17.5%) in the ICCT group received at least 1 psychiatrist visit (median, 3 visits; range, 1-9 visits) compared with 104 youths (82.5%) in the TAU group (median, 2.5 visits; range, 1–31 visits) (*P* < .001, χ^2^ test; φ = −0.67) (Table 4).

**Table 4.** Health Service Access and Utilization Among Youths and Caregivers by Treatment Group

Variable	ICCT group	TAU group	*P* value
OPOC-MHA, estimated marginal means (95% CI)			
Youths			
6 mo (n = 247)	3.37 (3.23-3.51)	3.27 (3.15-3.38)	.25^a^
12 mo (n = 218)	3.49 (3.34-3.64)	3.39 (3.23-3.55)	.35^a^
Caregiver			
6 mo (n = 189)	3.46 (3.32-3.60)	3.34 (3.20-3.49)	.24^a^
12 mo (n = 149)	3.61 (3.42-3.79)	3.40 (3.20-3.59)	.11^a^
Wait time (n = 212)			
No. of youths	105	107	<.001^b^
Median (IQR) [range], d	9 (5-16) [0-204]	27 (14-57) [0-283]	
Youths with ≥1 visit with a psychiatrist (n = 126)			
No. of youths	22	104	<.001^c^
No. of visits, median (range)	3 (1-9)	2.5 (1-31)	

Abbreviations: ICCT, integrated collaborative care team; OPOC-MHA, Ontario Perception of Care Tool for Mental Health and Addictions; TAU, treatment as usual.

^a^ Treatment-by-time interaction.

^b^ Two-sample *t* test.

^c^ χ^2^ test.

## Discussion

In collaboration with youths with lived MHSU experience and their caregivers, we developed and tested a new model of an IYS, the ICCT model. Among youths aged 14 to 17 years referred for hospital outpatient MHSU services, we did not observe differences between the ICCT and TAU in terms of change in functioning and most secondary measures. These findings were stable after accounting for age, gender, and study site. Within each group, youths and caregivers reported significant improvement in functioning and MHSU symptoms over time. There was no difference in positive service experiences. However, ICCT youths accessed services more rapidly and were less likely to see a psychiatrist. These latter 2 findings must be replicated and explored. It is possible that despite no differences in outcomes, the sooner youths receive services, the longer the sustained MHSU benefit. It is also possible that ICCT youths sought psychiatric support outside of their assigned site and that those in the TAU group accessed other MHSU services while waiting for hospital services. Cost-effectiveness needs to be assessed prior to making conclusions about resource efficiency.

Our findings that youths experienced improvement in functioning and MHSU measures within groups aligns with previous evaluations.^33,34,35^ The eDiscussion in Supplement 1 provides further details about these findings.

Codesigned with youths and families, this is the first rigorous pragmatic RCT (to our knowledge) to examine a specific integrated model of youth services systematically. Participants were successfully randomized, outcome assessments were conducted by blinded research staff, an intention-to-treat analysis was performed, there was excellent retention of participants, and there were no protocol deviations. Unfortunately, the COVID-19 pandemic halted recruitment, thus the trial may be underpowered.

ICCTs were intended to complement hospital MHSU services. Our results suggest that ICCTs may provide faster access to high-quality MHSU services and require less use of specialists, who tend to be a more costly resource. This is important, particularly given the overlap of ICCTs with IYSs^8^ and recent IYS spread across Canada. In Ontario, the ICCT model gave rise to Youth Wellness Hubs Ontario,^8^ one of Canada’s most established IYSs.^36^ The mental health policy question now is this: What place do integrated, community-based service models, like an IYS, hold in a comprehensive mental health system for youths? We suggest that IYSs work collaboratively alongside hospital services. One possibility is for a more comprehensive, integrated needs and MBC model, in which youths first access an IYS and, if needed, transition via a “warm handoff” to a hospital service. Of note, 117 of the 778 youths who were eligible for this study did not consent because they or their caregiver wished to access hospital outpatient treatment mostly to see a psychiatrist. Public acceptability of the IYS model is essential to ensure uptake.^7^

### Strengths and Limitations

Based on our experience, the ICCT model seems amenable to scale, given its ability to provide primary mental health care and its emphasis on allowing psychiatrists to focus on youths who need their services most.^8^ By emphasizing the roles of social workers, trained peer support workers, nurse practitioners, and others, our ICCT model is feasible for resource-limited communities, redirecting less severe needs away from psychiatrists and hospital services. Qualitative study findings from this project showed that ICCT youths reported positive service experiences (M. H. J. Quinlan-Davidson, PhD, unpublished data, 2024). Forthcoming articles on subgroup analyses and an economic evaluation will be critical to better understand the ICCT relative to TAU.

The study has several limitations, which are explained fully in the eDiscussion in Supplement 1 and summarized here. The most significant limitations include youths’ age range and the public health restrictions associated with the COVID-19 pandemic. Evaluating the ICCT model with youths of a broader age range will be critical in light of the unique needs of transition-aged youths^37,38^ and the recent push toward integrated broad-spectrum models of care for youths. TAU evolved over time to include some services delivered at the ICCT sites. Two secondary outcomes showed significant differences after adjusting for multiple comparisons. Well-developed measures of youth functioning are limited^39^; this study used the CIS despite documented limitations.^37,40^ Functioning was chosen by youths as the primary outcome, and the CIS seemed most appropriate at the time. This study ended prematurely due to COVID-19 public health restrictions^20^; consequently, it is underpowered, and important findings may not yet be evident. Improvement in each group may signify that both interventions are effective or that natural progression of MHSU conditions or regression to the mean has occurred.

## Conclusions

This pragmatic RCT provides evidence on an integrated model of service delivery in relation to hospital outpatient psychiatry services in youths aged 14 to 17 years. Given the high level of MHSU needs among youths, especially following the COVID-19 pandemic,^41,42^ as well as limited MHSU services and resources,^3,7^ it is critical to implement widely accessible, effective, and efficient youth MHSU services. These results provide a first step in establishing the evidence base for a new approach to MHSU service delivery, working in collaboration and integration with hospital outpatient services.

## Notes

Supplement 1. **eFigure 1.** Integrated Collaborative Care Team Model **eTable 1.** Guidance for Reporting Involvement of Patients and the Public 2.0 (GRIPP2)–Short Form **eTable 2.** Pragmatic Explanatory Continuum Indicator Summary (PRECIS-2) Scores for Trial Domains, Average Team Score
eMethods
**eTable 3**. Data Missingness Pattern for Columbia Impairment Scale (CIS) **eTable 4.** Demographic Characteristics of Complete and Incomplete Participants in Columbia Impairment Scale (CIS) Scores at T1 **eTable 5**. Frequency of Current Diagnoses (Diagnostic Interview for Affective and Anxiety Disorders–Child Version [DIAS-C]) in Both Treatment Arms (n = 233) **eTable 6.** Estimated Means and Pairwise Differences (95% CI) in Youth-Reported and Caregiver-Reported Youth Scores With Higher CIS and SDQ Scores Between and Within Treatment Arms (Complete Case Analysis) Across 3 Time Points
eResults
**eFigure 2.** Estimated Marginal Means (95% CI) in Youth- and Caregiver-Reported Youth Columbia Impairment Scale and Strengths and Difficulties Questionnaire Scores by Time Point and Treatment Arm, Unadjusted Models (n = 247 Youth, n = 189 Caregivers) **eFigure 3.** Estimated Marginal Means (95% CI) in Youths With Columbia Impairment Scale Scores by Time Point and Treatment Arm, Adjusted Model (n = 247)
eDiscussion

eReferences

Supplement 2.
Trial Protocol and Statistical Analysis Plan

Supplement 3.
Data Sharing Statement

## References

[1] Georgiades K, Duncan L, Wang L, Comeau J, Boyle MH; 2014 Ontario Child Health Study Team. Six-month prevalence of mental disorders and service contacts among children and youth in Ontario: evidence from the 2014 Ontario Child Health Study. Can J Psychiatry. 2019;64(4):246-255. doi:10.1177/0706743719830024 30978138 PMC6463361

[2] Settipani CA, Hawke LD, Virdo G, Yorke E, Mehra K, Henderson J. Social determinants of health among youth seeking substance use and mental health treatment. J Can Acad Child Adolesc Psychiatry. 2018;27(4):213-221. 30487936 PMC6254257

[3] Henderson JL, Cheung A, Cleverley K, . Integrated collaborative care teams to enhance service delivery to youth with mental health and substance use challenges: protocol for a pragmatic randomised controlled trial. BMJ Open. 2017;7(2):e014080. doi:10.1136/bmjopen-2016-014080 28167747 PMC5293997

[4] Patel V, Saxena S. Achieving universal health coverage for mental disorders. BMJ. 2019;366:l4516. doi:10.1136/bmj.l4516 31548204 PMC6753845

[5] Kieling C, Buchweitz C, Caye A, . Worldwide prevalence and disability from mental disorders across childhood and adolescence: evidence from the Global Burden of Disease Study. JAMA Psychiatry. 2024;81(4):347-356. doi:10.1001/jamapsychiatry.2023.5051 38294785 PMC10831630

[6] O’Reilly A, Illback R, Peiper N, O’Keeffe L, Clayton R. Youth engagement with an emerging Irish mental health early intervention programme (Jigsaw): participant characteristics and implications for service delivery. J Ment Health. 2015;24(5):283-288. doi:10.3109/09638237.2015.1019050 26191610

[7] McGorry PD, Mei C, Chanen A, Hodges C, Alvarez-Jimenez M, Killackey E. Designing and scaling up integrated youth mental health care. World Psychiatry. 2022;21(1):61-76. doi:10.1002/wps.20938 35015367 PMC8751571

[8] Henderson JL, Chiodo D, Varatharasan N, Andari S, Luce J, Wolfe J. Youth Wellness Hubs Ontario: development and initial implementation of integrated youth services in Ontario, Canada. Early Interv Psychiatry. 2023;17(1):107-114. doi:10.1111/eip.13315 35748798 PMC10084342

[9] Settipani CA, Hawke LD, Cleverley K, . Key attributes of integrated community-based youth service hubs for mental health: a scoping review. Int J Ment Health Syst. 2019;13(1):52. doi:10.1186/s13033-019-0306-7 31367230 PMC6651922

[10] Hetrick SE, Bailey AP, Smith KE, . Integrated (one-stop shop) youth health care: best available evidence and future directions. Med J Aust. 2017;207(10):S5-S18. doi:10.5694/mja17.00694 29129182

[11] Rickwood DJ, Telford NR, Mazzer KR, Parker AG, Tanti CJ, McGorry PD. The services provided to young people through the headspace centres across Australia. Med J Aust. 2015;202(10):533-536. doi:10.5694/mja14.01695 26021365

[12] McGorry PD, Tanti C, Stokes R, . headspace: Australia’s National Youth Mental Health Foundation—where young minds come first. Med J Aust. 2007;187(S7):S68-S70. doi:10.5694/j.1326-5377.2007.tb01342.x 17908032

[13] Greenhalgh T, Jackson C, Shaw S, Janamian T. Achieving research impact through co-creation in community-based health services: literature review and case study. Milbank Q. 2016;94(2):392-429. doi:10.1111/1468-0009.12197 27265562 PMC4911728

[14] Henderson J, Hess M, Mehra K, Hawke LD. From planning to implementation of the YouthCan IMPACT project: a formative evaluation. J Behav Health Serv Res. 2020;47(2):216-229. doi:10.1007/s11414-019-09658-4 31342279 PMC7231799

[15] Henderson JL, Hawke LD, Relihan J. Youth engagement in the YouthCan IMPACT trial. CMAJ. 2018;190(suppl):S10-S12. doi:10.1503/cmaj.180328 30404840 PMC6472455

[16] Henderson J, Courey L, Relihan J, . Youth and family members make meaningful contributions to a randomized-controlled trial: YouthCan IMPACT. Early Interv Psychiatry. 2022;16(6):670-677. doi:10.1111/eip.13232 34725926 PMC9544385

[17] Staniszewska S, Brett J, Simera I, . GRIPP2 reporting checklists: tools to improve reporting of patient and public involvement in research. Res Involv Engagem. 2017;3(1):13. doi:10.1186/s40900-017-0062-2 29062538 PMC5611595

[18] Heffernan OS, Herzog TM, Schiralli JE, Hawke LD, Chaim G, Henderson JL. Implementation of a youth-adult partnership model in youth mental health systems research: challenges and successes. Health Expect. 2017;20(6):1183-1188. doi:10.1111/hex.12554 28295940 PMC5689223

[19] Loudon K, Treweek S, Sullivan F, Donnan P, Thorpe KE, Zwarenstein M. The PRECIS-2 tool: designing trials that are fit for purpose. BMJ. 2015;350:h2147. doi:10.1136/bmj.h2147 25956159

[20] Information CIfH. COVID-19 Intervention Timeline in Canada. Canadian Institute for Health Information; 2021.

[21] Harris PA, Taylor R, Thielke R, Payne J, Gonzalez N, Conde JG. Research electronic data capture (REDCap)—a metadata-driven methodology and workflow process for providing translational research informatics support. J Biomed Inform. 2009;42(2):377-381. doi:10.1016/j.jbi.2008.08.010 18929686 PMC2700030

[22] Merikangas KR, Cui L, Heaton L, . Independence of familial transmission of mania and depression: results of the NIMH family study of affective spectrum disorders. Mol Psychiatry. 2014;19(2):214-219. doi:10.1038/mp.2013.116 24126930

[23] Edelbrock C, Bohnert A. Structured interviews for children and adolescents. In: Goldstein G, Hersen M, eds. Handbook of Psychological Assessment. 3rd ed. Pergamon; 2000:369-386. doi:10.1016/B978-008043645-6/50092-6

[24] Merikangas KR. NIMH Diagnostic Interview for Affective and Anxiety Spectrum Disorders–Child Version Procedures for Administration. National Institute of Mental Health; 2016.

[25] Bird HR, Shaffer D, Fisher P, . The Columbia Impairment Scale (CIS): pilot findings on a measure of global impairment for children and adolescents. Int J Methods Psychiatr Res. 1993;3(3):167-176.

[26] Goodman R. The Strengths and Difficulties Questionnaire: a research note. J Child Psychol Psychiatry. 1997;38(5):581-586. doi:10.1111/j.1469-7610.1997.tb01545.x 9255702

[27] Dennis ML, Feeney T, Stevens LH, Bedoya L. Global Appraisal of Individual Needs–Short Screener (GAIN-SS): Administration and Scoring Manual for the GAIN-SS Version 2.0.1. 2006. Chestnut Health Systems; 2008.

[28] Rush B, Lentinello E, Khalkhali S, . Development of a Client Perception of Care Tool for Mental Health and Addictions: Qualitative and Quantitative Psychometric Analysis. Centre for Addiction and Mental Health; 2014.

[29] McCoy CE. Understanding the intention-to-treat principle in randomized controlled trials. West J Emerg Med. 2017;18(6):1075-1078. doi:10.5811/westjem.2017.8.35985 29085540 PMC5654877

[30] SAS Institute. SAS Enterprise Miner 7.1 Reference Help. 2nd ed. SAS Institute; 2011.

[31] Tippey KGLM. An Ad Hoc Method for Computing Pseudo-Effect Size for Mixed Models. Texas A&M University; 2016.

[32] Fleiss JL, Levin B, Paik MC. Statistical Methods for Rates and Proportions. 3rd ed. John Wiley & Sons; 2003. doi:10.1002/0471445428

[33] Communio. Evaluation of Youth One Stop Shops. New Zealand Ministry of Health; 2009.

[34] O’Keeffe L, O’Reilly A, O’Brien G, Buckley R, Illback R. Description and outcome evaluation of Jigsaw: an emergent Irish mental health early intervention programme for young people. Ir J Psychol Med. 2015;32(1):71-77. doi:10.1017/ipm.2014.86 30185279

[35] Hilferty F, Cassells R, Muir K, . Is headspace Making a Difference to Young People’s Lives? Final Report of the Independent Evaluation of the headspace Program. Social Policy Research Centre, University of New South Wales; 2015.

[36] Fostering integrated care for young people in Canada. IYS home page. 2024. Accessed November 5, 2024. https://www.iys-sij.ca/

[37] Cleverley K, Davies J, Brennenstuhl S, . The Longitudinal Youth in Transition Study (LYiTS) cohort profile: exploration by hospital- versus community-based mental health services. Can J Psychiatry. 2022;67(12):928-938. doi:10.1177/07067437221115947 35924416 PMC9659798

[38] Hawke LD, Mehra K, Settipani C, . What makes mental health and substance use services youth friendly? A scoping review of literature. BMC Health Serv Res. 2019;19(1):257. doi:10.1186/s12913-019-4066-5 31029109 PMC6486969

[39] Barbic S, Brooks E, Lassak N, . “It cannot be boring!”: developing a measure of function for young adults accessing integrated youth services. J Patient Rep Outcomes. 2022;6(1):92. doi:10.1186/s41687-022-00491-6 36057736 PMC9440742

[40] Attell BK, Cappelli C, Manteuffel B, Li H. Measuring functional impairment in children and adolescents: psychometric properties of the Columbia Impairment Scale (CIS). Eval Health Prof. 2020;43(1):3-15. doi:10.1177/0163278718775797 29788789

[41] Racine N, McArthur BA, Cooke JE, Eirich R, Zhu J, Madigan S. Global prevalence of depressive and anxiety symptoms in children and adolescents during COVID-19: a meta-analysis. JAMA Pediatr. 2021;175(11):1142-1150. doi:10.1001/jamapediatrics.2021.2482 34369987 PMC8353576

[42] Blackwell CK, Mansolf M, Sherlock P, . Youth well-being during the COVID-19 pandemic. Pediatrics. 2022;149(4):e2021054754. doi:10.1542/peds.2021-054754 35301542 PMC9169239

