# Supplementary material for: Integrated Collaborative Care for Youths With Mental Health and Substance Use Challenges: A Randomized Clinical Trial
Source: JAMA Netw Open. 2025 May 13;8(5):e259565. doi: 10.1001/jamanetworkopen.2025.9565 (PMC12076176; doi:10.1001/jamanetworkopen.2025.9565)
Supplement: Supplement 1. — eFigure 1. Integrated Collaborative Care Team Model eTable 1. Guidance for Reporting Involvement of Patients and the Public 2.0 (GRIPP2)–Short Form eTable 2. Pragmatic Explanatory Continuum Indicator Summary (PRECIS-2) Scores for Trial Domains, Average Team Score eMethods eTable 3. Data Missingness Pattern for Columbia Impairment Scale (CIS) eTable 4. Demographic Characteristics of Complete and Incomplete Participants in Columbia Impairment Scale (CIS) Scores at T1 eTable 5. Frequency of Current Diagnoses (Diagnostic Interview for Affective and Anxiety Disorders–Child Version [DIAS-C]) in Both Treatment Arms (n = 233) eTable 6. Estimated Means and Pairwise Differences (95% CI) in Youth-Reported and Caregiver-Reported Youth Scores With Higher CIS and SDQ Scores Between and Within Treatment Arms (Complete Case Analysis) Across 3 Time Points eResults eFigure 2. Estimated Marginal Means (95% CI) in Youth- and Caregiver-Reported Youth Columbia Impairment Scale and Strengths and Difficulties Questionnaire Scores by Time Point and Treatment Arm, Unadjusted Models (n = 247 Youth, n = 189 Caregivers) eFigure 3. Estimated Marginal Means (95% CI) in Youths With Columbia Impairment Scale Scores by Time Point and Treatment Arm, Adjusted Model (n = 247) eDiscussion eReferences [file jamanetwopen-e259565-s001.pdf]

Supplementary Online Content

Henderson J, Szatmari P, Cleverley K, et al. Integrated collaborative care for youths with mental health and substance use challenges: a randomized clinical trial. *JAMA Netw Open*. 2025;8(5):e259565. doi:10.1001/jamanetworkopen.2025.9565

eFigure 1. Integrated Collaborative Care Team Model

eTable 1. Guidance for Reporting Involvement of Patients and the Public 2.0 (GRIPP2)–Short Form

eTable 2. Pragmatic Explanatory Continuum Indicator Summary (PRECIS-2) Scores for Trial Domains, Average Team Score

eMethods

eTable 3. Data Missingness Pattern for Columbia Impairment Scale (CIS)

eTable 4. Demographic Characteristics of Complete and Incomplete Participants in Columbia Impairment Scale (CIS) Scores at T1

eTable 5. Frequency of Current Diagnoses (Diagnostic Interview for Affective and Anxiety Disorders–Child Version [DIAS-C]) in Both Treatment Arms (n = 233)

eTable 6. Estimated Means and Pairwise Differences (95% CI) in Youth-Reported and Caregiver-Reported Youth Scores With Higher CIS and SDQ Scores Between and Within Treatment Arms (Complete Case Analysis) Across 3 Time Points

eResults

eFigure 2. Estimated Marginal Means (95% CI) in Youth- and Caregiver-Reported Youth Columbia Impairment Scale and Strengths and Difficulties Questionnaire Scores by Time Point and Treatment Arm, Unadjusted Models (n = 247 Youth, n = 189 Caregivers)

eFigure 3. Estimated Marginal Means (95% CI) in Youths With Columbia Impairment Scale Scores by Time Point and Treatment Arm, Adjusted Model (n = 247)

eDiscussion

eReferences

This supplementary material has been provided by the authors to give readers additional information about their work.

© 2025 Henderson J et al. *JAMA Network Open*.

eFigure 1. Integrated Collaborative Care Team Model

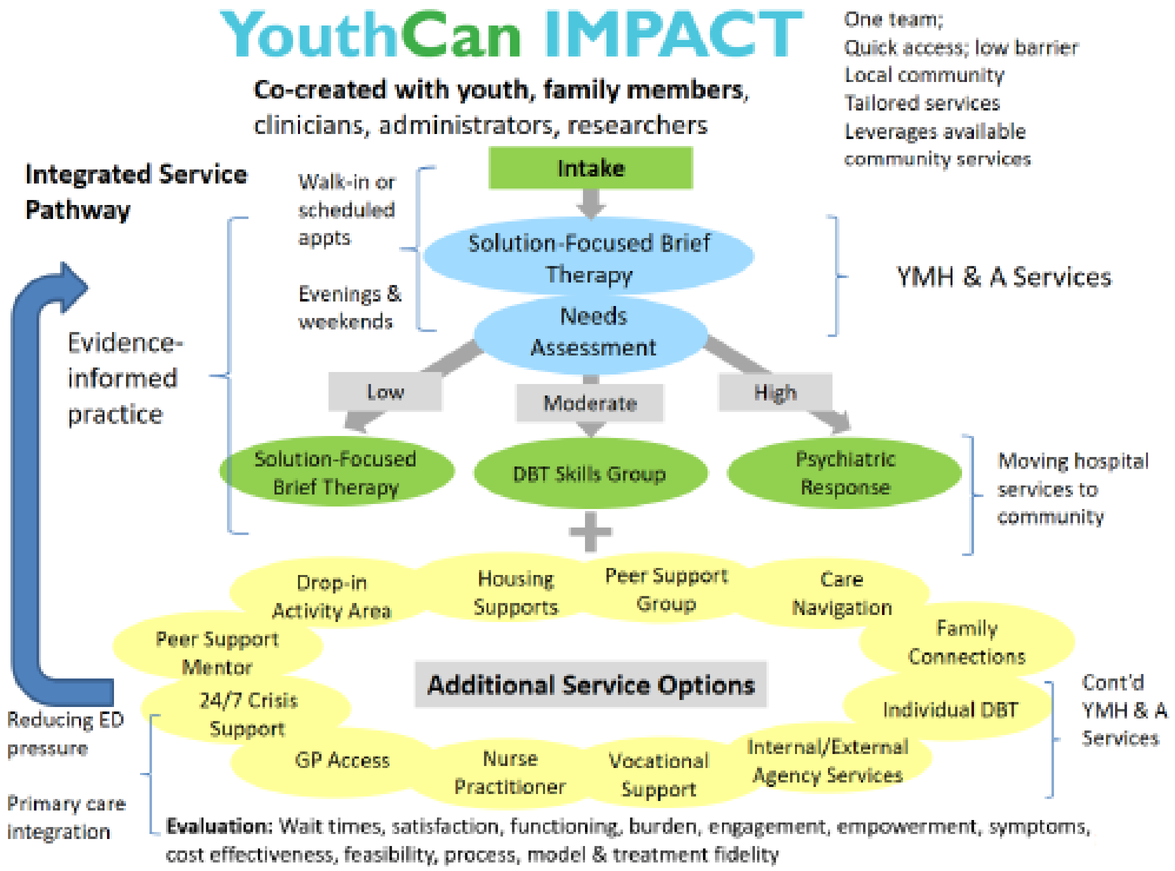

© 2025 Henderson J et al. *JAMA Network Open*.

eTable 1. Guidance for Reporting Involvement of Patients and the Public 2.0 (GRIPP2)–Short Form

**Section and topic**	**Item**	**Reported on page No**
1: Aim	To increase the relevance and appropriateness of the study, as well as ensure maximum study impact, we engaged youth and family members in the study conceptualization, design, implementation, and interpretation of the results.	4
2: Methods	Youth and family members were project team members, part of the project governance and members of the project's working groups. They helped define the study design by determining the primary outcome measure and contributing to the identification of secondary measures. They also contributed to designing the ICCT service pathway components. Youth and family members contributed to study implementation by training research staff on working with youth and family members, and ensuring that the research process was youth and family friendly.	7
3: Results	Youth and family members helped define the study design by determining the primary outcome measure and contributing to the identification of secondary measures. They also contributed to designing the ICCT service pathway components. Youth and family members have been co-authors on manuscripts and co-presenters of the research findings.	7
4: Discussion and Conclusions	This study was co-designed with youth and family members to rigorously examine the ICCT model using a pRCT. This initiative demonstrates the feasibility of youth, family members, community providers and researchers co-designing and implementing complex studies to address critical health challenges.	16
5: Reflections/ Critical Perspective	Engagement of youth and family members in study design and implementation has played a critical role in research and clinical service pathway design. Engaging youth and family members has improved the quality of research and service design and delivery.	16-19

© 2025 Henderson J et al. *JAMA Network Open*.

eTable 2. Pragmatic Explanatory Continuum Indicator Summary (PRECIS-2) Scores for Trial Domains, Average Team Score[1]

	Domain	Score	Rationale
1	Eligibility Criteria	3.8	Participants were recruited from usual entry process to hospital outpatient services. Tests were not used for selection. Most exclusions would apply for usual treatment.
2	Recruitment Path	4.2	Some extra effort invested for recruitment, in particular significant effort to remind usual intake staff of project. Participants compensated for completing measures once in the study and for travel.
3	Setting	3.6	Test intervention tested in purpose built settings to develop new usual care, so explanatory by design (Loudon et al 2015). Hospital recruitment sites and TAU sites all academic health sciences settings.
4	Organisation intervention	3.6	Training and use of measures/staging in ICCT is part of intervention but the trial is exploring how services are delivered so organizational change was required and should be considered explanatory (Loudon et al., 2015)
5	Flex of experimental intervention – Delivery	3.6	Measures and training in place as part of tested intervention but clinicians delivered care in accordance with usual practice
6	Flex of experimental intervention – Adherence	4.4	No specific measures were in place to enforce youth (patient) engagement in intervention or youth (patient) intervention adherence
7	Follow up	2.8	Baseline and follow-up measure completion at 6 and 12 months, plus participation in qualitative interviews for subset. Typically 3 extra visits.
8	Outcome	5	Outcome selected by youth (patients) and highly relevant to others.
9	Analysis	4.8	Intention to treat, no/minimal exclusions

Source: Loudon K, Treweek S, Sullivan F, Donnan P, Thorpe KE, Zwarenstein M. The PRECIS-2 tool: designing trials that are fit for purpose. BMJ. 2015 May 8;350:h2147. doi: 10.1136/bmj.h2147. PMID: 25956159.

[1]The Scores represent an average of the consensus ratings of five co-investigators completed after the trial was completed.

The mean score across all domains of the tool was 4.0 out of 5 indicating a high degree of pragmatic design.

© 2025 Henderson J et al. *JAMA Network Open*.

eMethods

Participants

Exclusion criteria included referral for primary diagnosis of an eating disorder; autism without MHSU problems; specialty forensic services; or need for immediate treatment due to imminent risk of self-harm or active psychosis. Potential participants were identified by hospital intake staff and referred to the study team. After a standardized screening call, interested youth and caregivers attended the referring hospital for an in-person session for where informed, signed consent; enrollment; baseline assessments; and randomization were conducted. Youth participants received $50 gift cards as honorarium at baseline (T1), and $75 gift cards at 6- and 12 months (T2 and T3). Caregivers received $25 gift cards at the same time points, and were entered into a random draw for a $250 gift card.

Randomization and Blinding.

Strata were defined based on sex at birth, as we assumed that anxiety and depression would be the most common MH disorders and these would show sex differences in prevalence[1, 2]. We were not sure these would be prognostic factors but did not want an imbalance by site in sex.

Measures and Instruments

Baseline characteristics.

Sociodemographics. Student status, birth place, ancestry/ethnic group/cultural background, and income were collected at intake. These sociodemographic factors were included as we followed studies using similar covariates from previously conducted youth RCTs[3].

Secondary Outcome Measures

Clinical Measures. The Strengths and Difficulties Questionnaire SDQ; [4] youth self-report and parental report was used as a secondary outcome. This 25-item questionnaire includes five

© 2025 Henderson J et al. *JAMA Network Open*.

subscales (emotional symptoms, conduct problems, hyperactivity/inattention, peer relationship problems, and prosocial behavior) with five-items in each subscale and a three-point Likert scale from "not true" to "certainly true." The total score was calculated by summing scores from all scales except the prosocial scale, with higher scores indicating greater difficulty.

The Global Appraisal of Individual Needs Short Screener (GAIN-SS) (version 3)-Substance Use Problems subscale [5] was used to screen youth for substance use disorders. Participants indicated whether they had experienced any of five substance use-related problems from "never" to "within the past month". For each participant, the GAIN-SS score was calculated as the number of problems present "within the past 2-3 months" or "within the past month". Prior research suggests that endorsement of three + substance use problems indicates a high likelihood of meeting diagnostic criteria for Substance Use Disorder and/or a need for services [5, 6]. As substance use problem scores were heavily skewed, we dichotomized the variable by classifying participants as having "no substance use problems in the last 3 months" and "any substance use problems in the last 3 months".

Service Experience. The Ontario Perception of Care Tool for Mental Health and Addictions, client and family/supporters versions OPOC; [7] was used to evaluate participants' perception and satisfaction with the services, including service strengths and areas for improvement. Youth completed the 23-item OPOC, while caregivers completed the 17-item family/supporters scale. Participants indicated whether they "strongly disagreed" to "strongly agreed" to statements about their service experiences, with higher scores indicating positive experiences and greater satisfaction with services.

Service Access and Utilization. This data was collected as part of the chart review and measured as wait time to a clinical intervention in the study and visits with a psychiatrist at both sites. Wait

© 2025 Henderson J et al. *JAMA Network Open*.

time was defined as date of randomization in the study to first clinical visit and measured in median number of days. Visits with a psychiatrist was measured as the proportion of youth who had at least one visit with the psychiatrist.

Statistical Analysis

We used mean imputation to impute missing items (or items rated as “not applicable”) from the outcome scales. If a measure was missing more than 50% of items, we omitted the observation from the analysis. We evaluated missingness for those who dropped out at the second or third time points compared to those with complete data. For the primary outcome, a two-sided alpha=0.05 was used. For secondary outcomes, to adjust for multiple comparisons, an alpha=0.005 was used.

Sensitivity analyses were performed for LMM and GEE models for youth participants, adjusting for gender identity (boy/man; girl/woman; and Trans, diverse gender identities), age at T1, and enrolling study site. Two-sided p-values <0.05 were considered statistically significant.

The analysis of secondary outcomes was exploratory, without adjustments for multiple hypotheses.

© 2025 Henderson J et al. *JAMA Network Open*.

eTable 3. Data Missingness Pattern for Columbia Impairment Scale (CIS) Score

		Number of missing items for CIS				
**Participant Type**	**Timepoint**	**0**	**1**	**3**	**13**	**Total**
Youth	T1	244	3	0	0	247
Youth	T2	215	0	0	32	247
Youth	T3	216	2	0	29	247
Caregiver	T1	186	3	0	0	189
Caregiver	T2	151	3	0	35	189
Caregiver	T3	146	2	1	40	189

© 2025 Henderson J et al. *JAMA Network Open*.

eTable 4. Demographic Characteristics of Complete and Incomplete Participants in Columbia Impairment Scale (CIS) Scores at T1

Youth - Variables	Complete (N=200)	Incomplete (N=47)
	**N (%)**	**N (%)**
**Age at enrollment**		
14	35 (17.6)	8 (17.0)
15	53 (26.6)	12 (25.5)
16	57 (28.6)	13 (27.7)
17	54 (27.1)	14 (29.8)
Missing	1 (0)	0 (0)
**Gender identity**		
Boy/Man	62 (31)	23 (48.9)
Girl/Woman	134 (67)	23 (48.9)
Trans, diverse gender identities, and missing	4 (2)	1 (2.1)
**Student status**		
Yes - Full time	192 (96)	44 (93.6)
Yes - Part time	5 (2.5)	1 (2.1)
No	3 (1.5)	2 (4.3)
**Born in Canada**		
No	25 (12.5)	10 (21.3)
Yes	175 (87.5)	37 (78.7)
**Ancestry/Ethnic group or cultural background**		
Another race/ethnicity[1]	6 (3)	1 (2.1)
Asian – East	6 (3)	3 (6.4)
Asian - South	20 (10)	3 (6.4)
Black	8 (4)	2 (4.3)
Don’t know	7 (3.5)	0 (0)
Indigenous (First Nations and Métis)	1 (0.5)	1 (2.1)
Latin American	14 (7.0)	5 (10.6)
Middle Eastern	3 (1.5)	2 (4.3)
Mixed Heritage	18 (9.0)	4 (8.5)
West Indian	5 (2.5)	1 (2.1)
White	112 (56.0)	25 (53.2)
**Caregiver - Variables**	**Complete (N=134)**	**Incomplete (N=55)**
	**Median (Range/N)**	**Median (Range/N)**
**Age at enrollment (in years)**	48 (20-63/n=128)	48 (31-83/n=55)
	**N (%)**	**N (%)**
Gender identity		
Boy/Man	12 (9.0)	7 (12.7)
Girl/Woman	121 (90.3)	48 (87.3)

© 2025 Henderson J et al. *JAMA Network Open*.

Caregiver - Variables	N (%)	N (%)
Trans, diverse gender identities, and missing	1 (0.75)	0 (0)
**Income**		
$0 to $29,999	16 (12.2)	13 (24.5)
$30,000 to $59,999	23 (17.6)	6 (11.3)
$60,000 to $89,999	12 (9.2)	1 (1.9)
$90,000 to $119,999	20 (15.3)	6 (11.3)
$120,000 to $149,999	17 (13.0)	8 (15.1)
$150,000 or over	43 (32.8)	19 (35.9)
Missing	3 (0)	2 (0)

[1]Another race/ethnicity subcategory was collected through open-ended responses and the following are listed as Canadian Jewish; Guyanese, St. Lucian; Mixed-Latin American and White North American; French Canadian, Italian, Métis, Scottish, South African; Greek; Spanish; White and South East Asian

eTable 5. Frequency of Current Diagnoses*(Diagnostic Interview for Affective and Anxiety Disorders–Child Version [DIAS-C]) in Both Treatment Arms (n = 233)

Disorder	Yes		No	
	**n**	**%**	**n**	**%**
Mood	174	74.7	59	25.3
Anxiety	200	85.9	33	14.2
ADHD	82	35.2	151	64.8
Disruptive Behaviour (CD and ODD)	39	16.7	194	83.3
OCD	36	15.5	197	84.5

*Mood disorders include major depressive disorder; Anxiety disorders include agoraphobia; generalized anxiety disorder; panic disorder; separation anxiety, social anxiety, general, social anxiety- performance disorders; ADHD=attention-deficit/hyperactivity disorder; Disruptive behaviour disorder includes conduct disorder and oppositional defiant disorder (ODD); and OCD=obsessive-compulsive disorder

Note that of the 233 participants with DIAS-C interviews (overall), 74.7%, 85.8%, and 35.2% of youth met the diagnostic threshold for any mood, any anxiety, and ADHD disorders, respectively

© 2025 Henderson J et al. *JAMA Network Open*.

eTable 6. Estimated Means and Pairwise Differences (95% CI) in Youth-Reported and Caregiver-Reported Youth Scores With Higher CIS and SDQ Scores Between and Within Treatment Arms (Complete Case Analysis) Across 3 Time Points [a]

			Estimated marginal means (95% CI)			Treatment-by-time Interaction p-value	Pairwise contrasts within arms	Pairwise contrasts between arms		
**Outcome**	**Sample Size (N)**	**Group**	**T1**	**T2**	**T3**		**Diff(T3-T1)**	**ICCT-TAU (T1)**	**ICCT-TAU (T2)**	**ICCT-TAU (T3)**
**CIS (Youth-reported) (unadjusted)**	156	**ICCT**	20.92 (19.64-22.20)	17.75 (16.47-19.03)	17.03 (15.75-18.31)	0.50	-3.36*** (-5.48- -2.31)	-0.54 (-1.31-2.38)	-0.35 (-2.19-1.50)	-0.81 (-2.65-1.03)
		**TAU**	20.39 (19.07-21.70)	18.10 (16.78-19.41)	17.84 (16.52-19.15)		-2.55** (-4.17- -0.92)			
**CIS (Youth-reported) – adjusted by age, site, gender identity**	155	**ICCT**	20.57 (18.44-22.70)	17.40 (15.27-19.53)	16.67 (14.55-18.80)	0.46	-3.48** (-5.23- -1.50)	0.53 (-1.33-2.40)	2.86 (1.00-4.73)	-0.91 (-2.78-0.95)
		**TAU**	20.04 (17.87-22.20)	17.71 (15.54-19.87)	17.59 (15.42-19.75)		-2.45** (-4.08- -0.81)			
**CIS (Caregiver-reported youth scores) (unadjusted)**	107	**ICCT**	19.81 (19.08-23.04)	17.45 (16.29-20.44)	16.57 (15.78-19.95)	0.31	-3.40** (-5.57- -1.23)	-0.16 (-2.33-2.01)	-1.77 (-3.94-0.40)	-2.12 (-4.29-0.048)

© 2025 Henderson J et al. *JAMA Network Open*.

			Estimated marginal means (95% CI)			Treatment-by-time Interaction p-value	Pairwise contrasts within arms	Pairwise contrasts between arms		
**Outcome**	**Sample Size (N)**	**Group**	**T1**	**T2**	**T3**		**Diff(T3-T1)**	**ICCT-TAU (T1)**	**ICCT-TAU (T2)**	**ICCT-TAU (T3)**
**CIS (Caregiver-reported youth scores) (unadjusted)**	**TAU**		19.97 (18.38-21.57)	19.21 (17.62-20.81)	18.70 (17.10-20.29)		-1.28 (-3.25-0.69)			
**CIS (Caregiver-reported youth scores) –adjusted by age, site, gender identity**	107	**ICCT**	20.90 (18.48 – 23.33)	18.54 (16.11 – 20.96)	17.66 (15.24 – 20.09)	0.31	-3.24** (-5.05- -1.43)	-0.28 (-2.49 – 1.93)	-1.89 (-4.10 – 0.32)	-2.24 (-4.45 – -0.033)
		**TAU**	21.18 (18.57 – 23.80)	20.42 (17.81 – 23.04)	19.91 (17.29 – 22.52)		-1.28 (-3.25-0.69)			
**SDQ (Youth-reported) (unadjusted)**	194	**ICCT**	20.22 (19.61-20.83)	18.79 (18.18-19.40)	18.83 (18.22-19.44)	0.67	-1.39** (-2.15- -0.62)	0.30 (-0.57-1.17)	0.49 (-0.38-1.35)	0.79 (-0.082-1.66)
		**TAU**	19.92 (19.31-20.54)	18.30 (17.69-18.92)	18.05 (17.43-18.66)		-1.88*** (-2.65- -1.10)			

© 2025 Henderson J et al. *JAMA Network Open*.

			Estimated marginal means (95% CI)			Treatment-by-time Interaction p-value	Pairwise contrasts within arms	Pairwise contrasts between arms		
**Outcome**	**Sample Size (N)**	**Group**	**T1**	**T2**	**T3**		**Diff(T3-T1)**	**ICCT-TAU (T1)**	**ICCT-TAU (T2)**	**ICCT-TAU (T3)**
**SDQ (Youth-reported) – adjusted by age, site, gender identity**	193	**ICCT**	20.00 (19.01 – 20.99)	18.57 (17.58 – 19.56)	18.61 (17.62 – 19.60)	0.63	-1.39** (-2.15- - 0.62)	0.30 (-0.57- 1.18)	0.51 (-0.37- 1.39)	0.83 (-0.045- 1.71)
		**TAU**	19.69 (18.69 – 20.70)	18.06 (17.06 – 19.07)	17.78 (16.77 – 18.79)		-1.92*** (-2.69- - 1.14)			
**SDQ (Caregiver-reported youth scores) (unadjusted)**	131	**ICCT**	18.77 (18.03- 19.51)	17.28 (16.54- 18.01)	16.86 (16.13- 17.85)	0.96	-1.91*** (-2.85- - 0.97)	0.10 (-0.93- 1.14)	0.16 (-0.88- 1.19)	-0.03 (-1.07- 1.00)
		**TAU**	18.67 (17.94- 19.40)	17.12 (16.39- 17.85)	16.89 (16.16- 17.62)		-1.77** (-2.71- - 0.83)			
**SDQ (Caregiver – reported youth scores) – adjusted by age, site, gender identity**	131	**ICCT**	18.84 (17.77 – 19.90)	17.35 (16.28– 18.41)	16.93 (15.87 – 18.00)	0.96	-1.91*** (-2.85- - 0.97)	0.074 (-0.97- 1.12)	0.13 (-0.92- 1.17)	-0.061 (-1.11- 0.98)
		**TAU**	18.76 (17.63 – 19.89)	17.22 (16.09 – 18.35)	16.99 (15.86 – 18.12)		-1.77** (-2.71- - 0.84)			

© 2025 Henderson J et al. *JAMA Network Open*.

[a] Estimated marginal means for adjusted models assume the average value of model covariates, *<0.05 **<0.01 ***<0.001

© 2025 Henderson J et al. *JAMA Network Open*.

eResults

Sample Description

The two most common reasons for non-participation were that the potential participants could not be contacted, or they preferred to access hospital treatment.

Columbia Impairment Scale – Caregiver-reported

Pairwise contrasts of the unadjusted model showed that ICCT caregivers at T3 scored youth significantly lower compared to T1 (d=-3.21; 95% CI=[-4.77, -1.65], p<0.0001). Similarly, TAU caregivers at T3 provided significantly lower mean CIS scores compared to T1 (d=-2.67; 95% CI=[-4.35, -1.18], p=0.0007) (Table 2) (eFigure 3) indicating improved functioning in both arms. There were no significant differences in scores between ICCT and TAU across the three time points (treatment arm-by-time interaction p=0.75; η^2=0.002) in the unadjusted model.

Strengths and Difficulties Questionnaire

ICCT youth at T3 had significantly lower mean total SDQ scores compared to T1 (d=-1.74; 95% CI=[-2.58, -0.90], p<0.0001). Similarly, TAU youth at T3 had significantly lower mean SDQ scores compared to T1 (d=-1.78; 95% CI=[-2.63, -0.93], p<0.0001) (Table 2) (eFigure 3) indicating a reduction in psychopathology scores across time in both arms.

There were no significant differences in mean SDQ scores between youth in ICCT and TAU across the three time points in the unadjusted model (treatment arm-by-time interaction p=0.84) or adjusted model (p=0.86). This was also true for caregiver-reported youth scores (treatment arm-by-time interaction p=0.74).

Ontario Perception of Care Tool for Mental Health and Addictions

© 2025 Henderson J et al. *JAMA Network Open*.

For youth, there were no significant differences between treatment arms in youth-reported OPOC scores at either T2 (ICCT: mean=3.37 [95% CI=(3.23, 3.51)] vs TAU: 3.27, [(3.15, 3.38)]; pooled t-test *p*=0.25) or T3 (ICCT: 3.49 [(3.34, 3.64)] vs TAU: 3.39 [(3.23, 3.55)]; *p*=0.35). Similarly, there was no difference in OPOC scores as reported by caregivers at T2 (ICCT: 3.46, [(3.32, 3.60)] vs TAU: 3.34 [(3.20, 3.49)]; *p*=0.24) or T3 (ICCT: 3.61 [(3.42, 3.79)] vs TAU: 3.40 [(3.20, 3.59)]; *p*=0.11) (Table 4). Both groups had mean scores within the positive treatment/support experiences and 'satisfaction with services'[8] range.

© 2025 Henderson J et al. *JAMA Network Open*.

eFigure 2. Estimated Marginal Means (95% CI) in Youth- and Caregiver-Reported Youth Columbia Impairment Scale and Strengths and Difficulties Questionnaire Scores by Time Point and Treatment Arm, Unadjusted Models (n = 247 Youth, n = 189 Caregivers)

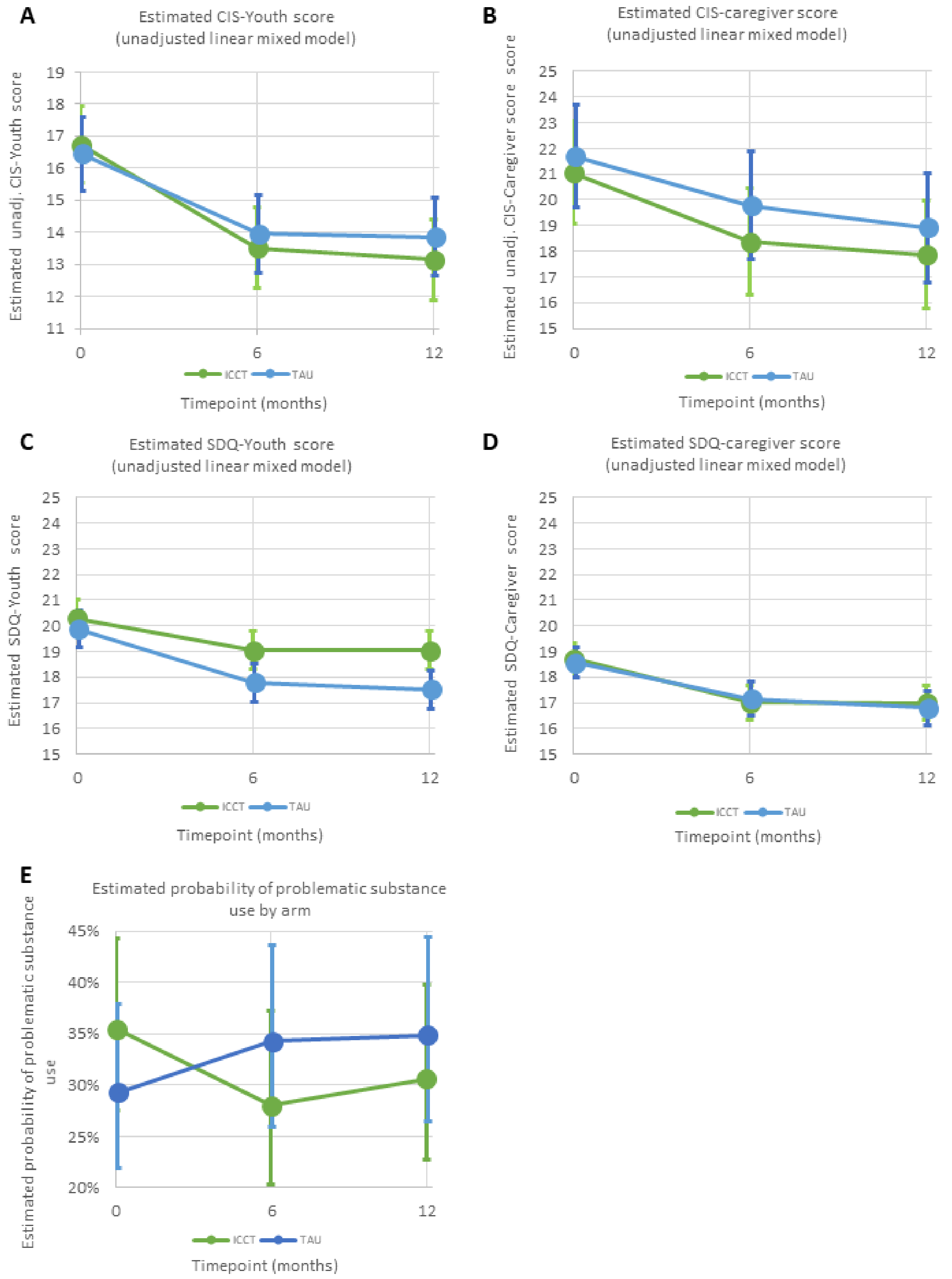

© 2025 Henderson J et al. *JAMA Network Open*.

eFigure 3. Estimated Marginal Means (95% CI) in Youths With Columbia Impairment Scale Scores by Time Point and Treatment Arm, Adjusted Model (n = 247)

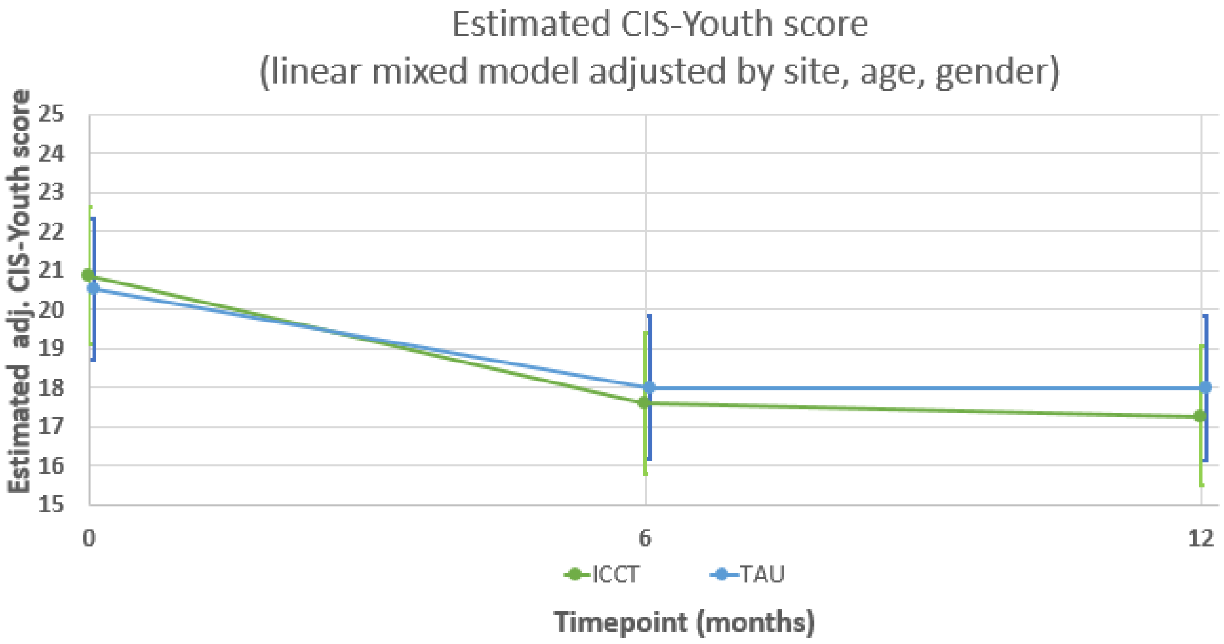

eDiscussion

94% of youth using New Zealand's Youth One Stop Shops[9] reported improved health. Similar results were reported in an evaluation of Ireland's Jigsaw services[10], and Australia's headspace[11]. Although we did show within-arm statistical significance on the CIS in both the ICCT and TAU, further research needs to be conducted on the clinical significance of these findings[12], including qualitative research to explore youth's perception of improvement in mental health conditions.

It is possible that youth may have been satisfied to receive any service. Although satisfaction may be difficult to assess due to variations in how it is defined and experienced [13], we used the OPOC, a validated measure of youth perception on their experience of care [8].

© 2025 Henderson J et al. *JAMA Network Open*.

Further research to understand youth satisfaction in services and develop a standard definition of satisfaction is warranted.

Our sample was generally comparable to the overall youth population in our community. According to the 2016 Toronto Census, 1.3% of the entire Toronto population (regardless of age) identified as Indigenous (First Nations and Métis) and 50.7% as Black and/or racialized [14]. In the current study, 0.8% of youth identified as Indigenous and 43.7% as Black and/or racialized. Our sample was not generally comparable to the population of Toronto based on place of birth. In 2016, there were approximately 46.3% of individuals born outside of Canada, while the current study included 14.2% of youth who were born outside of Canada.

ICCT and TAU were not effective at reducing the probability of screening positive for a substance use disorder (SUD). Given that SUDs are widespread among youth [15, 16, 17] and a risk factor for mental health concerns [18], greater research needs to be conducted on interventions addressing SUD among young people.

MBC is an essential ingredient of an integrated system to ensure that youth who have not responded to treatment, are at risk of psychosis, or imminent risk of serious self-harm can be fast-tracked to more intensive hospital services[19].

Limitations.

For example, group DBT became instituted as a treatment modality at two hospitals, leading to some contamination. Within complex interventions, such services are difficult to confine. This contamination could have diluted the treatment contrast and could have biased the estimate of the effectiveness of the intervention [20].

© 2025 Henderson J et al. *JAMA Network Open*.

Although the proportion of participants who declined to participate, among participants assessed for eligibility, is in in line with RCT refusal rates[21], it would be important to conduct further research to determine why participants decline to participate in RCTs and/or consider randomization to innovative treatments in mental health. Following the pRCT design, we tried to mimic real-world services as much as possible; as such, young people were not blinded to the treatment arm (it would be impossible to do so in any case). Knowledge of treatment arm assignment could have affected their behaviour in the study and response to outcome measures. This knowledge is a common problem with complex interventions that are based on psychosocial interventions. Outcome measures were self-reported by youth and caregivers. As such, there could have been response bias in terms of providing biased assessments of their behaviour and the effectiveness of the intervention. There were a number of delays in launching the study, including delays from the negotiating partnership and legal agreements, obtaining ethics approvals, and embedding research processes into existing practices[22] across participating hospitals and community organizations. In addition, the study experienced several recruitment challenges. As the pandemic progressed, the rate of recruitment slowed. At the same time, we wanted to ensure that we were able to analyze the data fulsomely before funding deadlines. The study also experienced delays to recruitment due to the multi-site and multi-recruitment pathway nature of the trial. Being underpowered could have negatively impacted the likelihood of a statistically significant finding reflecting a true effect and the magnitude of that effect could have been inflated relative to the true effect. It is possible that youth experienced improved functioning among those who did not receive any treatment. This could have been due to passage of time, having been seen at hospital intake, or youth could have been receiving services outside of the remit of the study.

© 2025 Henderson J et al. *JAMA Network Open*.

eReferences

1. Riecher-Rössler A. Prospects for the classification of mental disorders in women. Eur Psychiatry. 2010;25(4):189-96
2. Seedat S, Scott KM, Angermeyer MC, Berglund P, Bromet EJ, Brugha TS, et al. Cross-national associations between gender and mental disorders in the World Health Organization World Mental Health Surveys. Arch Gen Psychiatry. 2009;66(7):785-95
3. Richardson LP, Ludman E, McCauley E, Lindenbaum J, Larison C, Zhou C, et al. Collaborative care for adolescents with depression in primary care: a randomized clinical trial. Jama. 2014;312(8):809-16
4. Goodman R. The Strengths and Difficulties Questionnaire: A research note. Child Psychology & Psychiatry & Allied Disciplines. 1997;38(5):581-6
5. Dennis ML, Feeney, T., Stevens, L. H. . Global Appraisal of Individual Needs–Short Screener (GAIN-SS): Administration and Scoring Manual for the GAINSS Version 2.0.1. Bloomington, IL: Chestnut Health Systems; 2006.
6. Dennis ML, Chan YF, Funk RR. Development and validation of the GAIN Short Screener (GSS) for internalizing, externalizing and substance use disorders and crime/violence problems among adolescents and adults. Am J Addict. 2006;15 Suppl 1(Suppl 1):80-91
7. Rush B, Hansson E, Cvetanova Y, Rotondi N, Furlong A, Behrooz R. Development of a client perception of care tool for mental health and addictions: Qualitative, quantitative, and psychometric analysis. Final Report for the Ministry of Health and Long-Term Care. Toronto, Ontario: Health Systems and Health Equity Research, Centre for Addiction and Mental Health; 2013.
8. Rush B L, E, Khalkhali, S, Cvetanova, Y, Rotondi, N, Furlong, A, Chau N, , Behrooz R. Development of a Client Perception of Care Tool for Mental Health and Addictions: Qualitative and Quantitative Psychometric Analysis Toronto: Centre for Addiction and Mental Health; 2014.
9. Communio. Evaluation of Youth One Stop Shops. Wellington: New Zealand Ministry of Health; 2009.
10. O'Keeffe L, O'Reilly A, O'Brien G, Buckley R, Illback R. Description and outcome evaluation of Jigsaw: an emergent Irish mental health early intervention programme for young people. Ir J Psychol Med. 2015;32(1):71-7
11. Hilferty F, Cassells, R., Muir, K., Duncan, A., Christensen, D.,, Mitrou F, Gao, G., Mavisakalyan, A., Hafekost, K., Tarverdi,, Y. N, H., Wingrove, C. and Katz, I. Is headspace making a difference to young people's lives? Final Report of the independent evaluation of the headspace program. Sydney: Social Policy Research Centre, UNSW Australia.; 2015.
12. Krause KR, Lee A, Shan D, Cost KT, Hawke LD, Cheung AH, et al. Minimally important change on the Columbia Impairment Scale and Strengths and Difficulties Questionnaire in youths seeking mental healthcare. BMJ Ment Health. 2025;28(1)
13. Carretta E, Bond TG, Cappiello G, Fantini MP. Looking Through the Patients' Eyes: Measuring Patient Satisfaction in a Public Hospital. J Patient Exp. 2017;4(3):121-8
14. Census Profile, 2016 Census Toronto, City [Census subdivision], Ontario and Canada [Country] [Internet]. Statistics Canada. Available from: https://www12.statcan.gc.ca/census-recensement/2016/dp-pd/prof/details/Page.cfm?Lang=E&Geo1=CSD&Code1=3520005&Geo2=PR&Data=Count&B1=All
15. Kieling C, Buchweitz C, Caye A, Silvani J, Ameis SH, Brunoni AR, et al. Worldwide Prevalence and Disability From Mental Disorders Across Childhood and Adolescence: Evidence From the Global Burden of Disease Study. JAMA Psychiatry. 2024

© 2025 Henderson J et al. *JAMA Network Open*.

16. Pearson CJ, T.; Ali, J. Health at a Glance: Mental and Substance Use Disorders in Canada. Ottawa, ON: Statistics Canada; 2013.
17. Aderibigbe OO, Stewart SL, Hirdes JP, Perlman C. Substance Use among Youth in Community and Residential Mental Health Care Facilities in Ontario, Canada. Int J Environ Res Public Health. 2022;19(3)
18. Esmaeelzadeh S, Moraros J, Thorpe L, Bird Y. Examining the Association and Directionality between Mental Health Disorders and Substance Use among Adolescents and Young Adults in the U.S. and Canada-A Systematic Review and Meta-Analysis. J Clin Med. 2018;7(12)
19. Lewis CC, Boyd M, Puspitasari A, Navarro E, Howard J, Kassab H, et al. Implementing Measurement-Based Care in Behavioral Health: A Review. JAMA Psychiatry. 2019;76(3):324-35
20. Magill N, Knight R, McCrone P, Ismail K, Landau S. A scoping review of the problems and solutions associated with contamination in trials of complex interventions in mental health. BMC Medical Research Methodology. 2019;19(1):4
21. Lin LY, Jochym N, Merz JF. Refusal rates and waivers of informed consent in pragmatic and comparative effectiveness RCTs: A systematic review. Contemp Clin Trials. 2021;104:106361
22. Hawke LD, Mehra K, Settipani C, Relihan J, Darnay K, Chaim G, et al. What makes mental health and substance use services youth friendly? A scoping review of literature. BMC Health Serv Res. 2019;19(1):257

© 2025 Henderson J et al. *JAMA Network Open*.
