# Supplementary material for: Integrated Collaborative Care for Youths With Mental Health and Substance Use Challenges: A Randomized Clinical Trial
Source: JAMA Netw Open. 2025 May 13;8(5):e259565. doi: 10.1001/jamanetworkopen.2025.9565 (PMC12076176; doi:10.1001/jamanetworkopen.2025.9565)
Supplement: Supplement 2. — Trial Protocol and Statistical Analysis Plan [file jamanetwopen-e259565-s002.pdf]

ClinicalTrials.gov PRS
Protocol Registration and Results System

ID: #012/2016 Integrated Collaborative Care Teams for Youth With Mental Health and/or Addiction Challenges (YouthCan IMPACT) NCT02836080

Protocol Registration Preview

This is a rough approximation of how the Protocol Registration will appear on the ClinicalTrials.gov public web site.

Integrated Collaborative Care Teams for Youth With Mental Health and/or Addiction Challenges (YouthCan IMPACT)

The safety and scientific validity of this study is the responsibility of the study sponsor and investigators. Listing a study does not mean it has been evaluated by the U.S. Federal Government. Read our disclaimer for details.

ClinicalTrials.gov Identifier: NCT02836080

Recruitment Status: Active, not recruiting
First Posted: *
Last Update Posted: *

* Date not available in PRS

Sponsor:

Centre for Addiction and Mental Health

Collaborators:

East Metro Youth Services
Delisle Youth Services
LOFT Community Services
The Sashbear Foundation
The Anne Johnston Health Station

South East Toronto Family Health Team
Institute for Clinical Evaluative Sciences
Medical Psychiatry Alliance
Graham Boeckh Foundation
The Ontario Spor Support Unit

Information provided by (Responsible Party):

Joanna Henderson, Centre for Addiction and Mental Health

Study Description

Brief Summary:

Among youth, the prevalence of mental health and addiction (MHA) disorders is roughly 20%, yet youth are challenged to access services in a timely fashion. To address MHA system gaps, this study will test the benefits of an Integrated Collaborative Care Team (ICCT) model for at-risk youth with MHA challenges. In partnership with community agencies, adolescent psychiatry hospital departments, and family health teams, investigators have developed an innovative model of service provision involving rapid access to MHA services. This model will be implemented and compared to the usual treatment youth receive in hospital-based, outpatient, mental health clinics in Toronto. A rapid, systematic, approach to MHA services geared to need in a youth-friendly environment is expected to result in better MHA outcomes for youth. Moreover, the ICCT approach is expected to decrease service wait-times, be more youth- and family-centred, and be more cost-effective.

Condition or disease	Intervention/treatment
Mental Disorders<br>Addiction	Behavioral: Integrated Collaborative Care Team (ICCT)<br>Treatment as Usual (TAU)

Detailed Description:

This study is a pragmatic randomized control trial (RCT) with random allocation occurring within each hospital site to either treatment as usual (TAU) (4 out-patient hospital sites) or treatment at one of 3 community-based Integrated Collaborative Care Teams (ICCTs). A total of 500 youth aged 14-18 with mental health and/or addictions (MHA) concerns, referred for out-patient services at one of four local hospitals, will be randomized to receive ICCT care versus TAU. For each youth, a primary caregiver will also be recruited into the study, if available.

With wide inclusion criteria and a design meant to emulate a "real world" setting, this study will rigorously evaluate a service delivery model composed of multiple interventions for youth presenting with a broad range of MHA problems. The ICCT is expected to result in better MHA outcomes, show better performance indicators, and be more cost-effective than TAU.

Study Design

Study Type:	Interventional
Actual Enrollment:	247 participants
Allocation:	Randomized
Intervention Model:	Parallel Assignment
Masking:	Single (Investigator)
Primary Purpose:	Treatment
Official Title:	Integrated Collaborative Care Teams to Enhance Service Delivery to Youth With Mental Health and Substance Use Challenges: Protocol for a Pragmatic Randomized-controlled Trial
Actual Study Start Date:	September 2016
Actual Primary Completion Date:	April 2021
Estimated Study Completion Date:	April 2025

Arms and Interventions

Arm	Intervention/treatment
Experimental: Integrated Collaborative Care Team<br><br>Integrated Collaborative Care Team (ICCTs) are housed in the local community to improve youth access, in three neighborhoods across Toronto (East Metro Youth Services [EMYS]-Scarborough, EMYS-Southeast Toronto, and Delisle Youth Services-Central Toronto). Each ICCT will include a variety of service providers and coordinated patient care delivering evidence-informed interventions in a stepped-care model.	Behavioral: Integrated Collaborative Care Team (ICCT)<br><br>An integrated, collaborative pathway of needs-based services. ICCTs will offer a wide variety of services, including Solution-Focused Brief Therapy (SFBT) on a scheduled and walk-in basis, care navigators, various clinician-guided interventions, psychiatry, nurse practitioner services, access to primary care, and peer support, all co-located in youth-friendly, community-based clinics. For each intervention, standardized intervention protocols will be used.
Active Comparator: Treatment as Usual (TAU)<br><br>The comparator arm consists of out-patient TAU in a hospital setting and will occur at one of four outpatient hospital sites across Toronto.<br><br>Partners include the following four hospitals: Hospital for Sick Children (SickKids), the Centre for Addiction and Mental Health (CAMH), Michael Garron Hospital (formerly the Toronto	Treatment as Usual (TAU)<br><br>Standard out-patient treatment provided at each participating hospital site. This typically entails referral to a psychiatrist at the participating hospital, who will provide assessment and treatment, with referral to appropriate services, guided by local treatment protocols.

East General Hospital), and Sunnybrook Hospital.	

Outcome Measures

Primary Outcome Measure:

1. Youth functioning [Time Frame: One year]

   Measured using the Columbia Impairment Scale (CIS)

Secondary Outcome Measures:

1. Clinical improvement [Time Frame: One year]

   Measured using Strengths and Difficulties Questionnaire

2. Problematic substance use [Time Frame: One year]

   Assessed using the GAIN Short Screener and the substance use table of the Adolescent Alcohol and Drug Involvement Scale

3. Satisfaction with the service models [Time Frame: One year]

   Assessed using the Ontario Perception of Care Tool for Mental Health and Addictions

4. Continuity of care [Time Frame: One year]

   Measured using the Continuity of Care in Children's Mental Health questionnaire

5. Goal attainment [Time Frame: One year]

   Measured using a custom form indicating goals established by the youth and caregiver at intake, followed by a rating of goal attainment

6. Client empowerment and engagement [Time Frame: One year]

   Measured using the Family Empowerment Scale for caregivers, and the Youth Efficacy/Empowerment Scale for youth

7. Caregiver burden [Time Frame: One year]

   Measured using the Burden Assessment Scale

8. Quality-adjusted life years (QALYs) [Time Frame: One year]

Measured using the Assessment of Quality of Life-6D (AQOL-6D)

9. Cost-effective analysis (CEA) and a cost-utility analysis (CUA) [Time Frame: One year]

   Incremental costs of ICCT compared to TAU (treatment as usual) in improving health outcomes

Eligibility Criteria

Ages Eligible for Study: 14 Years to 18 Years
Sexes Eligible for Study: All
Accepts Healthy Volunteers: No

Criteria

Inclusion Criteria:

1. Provision of informed consent
2. Aged 14 - 17 years 11 months
3. New referrals to one of the four participating hospitals for out-patient MHA services
4. Among the population regularly accepted for out-patient services at that hospital

Exclusion Criteria:

1. Referral for specialty forensic or firesetting treatment
2. Moderate to severe intellectual disability or autism without MHA problems
3. Primary diagnosis of an eating disorder
4. Active psychosis or imminent risk of self-harm requiring immediate intervention
5. Inability to read and write English or to consent to the study

Contacts and Locations

Locations

Canada, Ontario

Centre for Addiction and Mental Health (CAMH)
Toronto, Ontario, Canada, M6J 1H4
Sunnybrook Hospital
Toronto, Ontario, Canada, M4N 3M5
Michael Garron Hospital
Toronto, Ontario, Canada, M4C 3E7
The Hospital for Sick Children
Toronto, Ontario, Canada, M5G 1X8
North York General Hospital

Toronto, Ontario, Canada, M2K 1E1

Investigators

Principal Investigator:	Joanna Henderson, Ph.D., C.Psych	Director, Margaret and Wallace McCain Centre for Child, Youth and Family Mental Health; Clinician Scientist, Centre for Addiction and Mental Health; Associate Professor, Dept. of Psychiatry, University of Toronto
Principal Investigator:	Peter Szatmari, MD, FRCPC	Chief, Child and Youth Mental Health Collaborative, The Hospital for Sick Children and Centre for Addiction and Mental Health; Professor and Head of the Division of Child and Youth Mental Health, University of Toronto
Principal Investigator:	Amy Cheung, MD, FRCPC	Associate Scientist, Evaluative Clinical Sciences, Hurvitz Brain Sciences Research Program, Sunnybrook Research Institute; Associate Professor, Department of Psychiatry, University of Toronto
Principal Investigator:	Kristin Cleverley, RN, Ph.D.	CAMH Chair in Mental Health Nursing Research; Assistant Professor, University of Toronto; Clinician-Scientist, Margaret and Wallace McCain Centre for Child, Youth & Family Mental Health, Centre for Addiction and Mental Health
Principal Investigator:	Gloria Chaim, M.S.W.	Associate Director, Child Youth and Family Services; Head, Community Engagement and Partnership, Margaret and Wallace McCain Centre for Child, Youth and Family Mental Health, CAMH; Assistant Professor, Dept. of Psychiatry, University of Toronto

More Information

Other Resources:

Links provided by Joanna Henderson, Centre for Addiction and Mental Health

Information about research at the Centre for Addiction and Mental Health, Canada's largest mental health and addiction teaching hospital

Responsible Party:	Joanna Henderson, Director, Margaret and Wallace McCain Centre for Child, Youth and Family Mental Health; Associate Professor of Psychiatry, University of Toronto, Centre for Addiction and Mental Health
ClinicalTrials.gov Identifier:	NCT02836080
Other Study ID Numbers:	#012/2016

Last Verified: August 2023

Individual Participant Data (IPD) Sharing Statement:

Plan to Share IPD: Undecided

Human Subjects Protection Review Board Status: Approved

U.S. National Library of Medicine | U.S. National Institutes of Health | U.S. Department of Health & Human Services | HHS Vulnerability Disclosure

YouthCAN IMPACT randomized controlled trial

Statistical Analysis Plan

Joanna L Henderson, Amy Cheung, Kristin Cleverley, Gloria Chaim, Myla E Moretti, Claire de Oliveira, Lisa D Hawke, Andrew R Willan, Di Shan, Clement Ma, David O'Brien, Olivia Heffernan, Tyson Herzog, Lynn Courey, Heather McDonald, Enid Grant, Peter Szatmari

September 1, 2022

Study objectives

Primary objectives

1. To test the benefits of an Integrated Collaborative Care Team (ICCT) model in improving functioning among youth aged 14–18 years with MHA challenges, compared to TAU.
2. To test the benefits of an Integrated Collaborative Care Team (ICCT) model in improving functioning among family members with a youth aged 14–18 years with MHA challenges, compared to TAU.

Secondary objectives

1. To test the benefits of an Integrated Collaborative Care Team (ICCT) model in improving symptoms among youth aged 14–18 years with MHA challenges, compared to TAU.
2. To test the benefits of an Integrated Collaborative Care Team (ICCT) model in improving symptoms among family members with a youth aged 14–18 years with MHA challenges, compared to TAU.
3. To compare the service experience of ICCT versus TAU at 12-months post-randomization (T3) among youth aged 14-18 years with MHA challenges.
4. To compare the service experience of ICCT versus TAU at 12-months post-randomization (T3) among family members with a youth aged 14-18 years with MHA challenges.
5. To test the benefits of an Integrated Collaborative Care Team (ICCT) model in improving substance use among youth aged 14–18 years with MHA challenges, compared to TAU.

Study endpoints

Bolded endpoints will be analyzed for the primary analysis paper.

Primary endpoints

1. **Youth-reported Columbia Impairment Scale (CIS) measured at baseline, 6 months, and 12 months post-randomization.**
2. **Family member-reported Columbia Impairment Scale (CIS) measured at baseline, 6 months, and 12 months post-randomization.**

Secondary endpoints

1. Symptoms:
    a. **Strengths and Difficulties (Youth & Caregiver; T1, T2, T3)**

    b. DIAS-C (Youth & Caregiver; T1) - descriptive
    c. PRIME (Youth; T1)
    d. PCL-C (Youth; T1)
2. Burden
    a. Burden Assessment Scale (Caregiver; T1, T2, T3)
3. Service Experience
    a. **Ontario Perception of Care Tool for Mental Health and Addictions (Youth; T2, T3)**
    b. **Ontario Perception of Care Tool for Mental Health and Addictions – Family (Caregiver; T2, T3)**
4. Physical and sexual health needs
    a. Custom Physical Health Scale (Youth & Caregiver; T1)
5. Substance use
    a. **GAIN-SS (Youth; T1, T2, T3)**
    b. AADIS (Youth; T1, T2, T3)
6. Demographics (Youth & Parent; T1, T2, T3)
7. Utility Measure
    a. Assessment of Quality of Life-6D (Youth; T1)
8. Empowerment/Engagement
    a. Youth Efficacy/ Empowerment Scale (Youth; T1, T2, T3)
    b. Family Empowerment Scale (Caregiver; T1, T2, T3)
9. Continuity of Care
    a. Continuity of Care in Children's Mental Health (Youth & Caregiver; T1, T2, T3)
10. Goals
    a. Goal progress chart/custom question (Youth & Caregiver; T1, T2, T3)
11. Economic Evaluation [To be analyzed by the health economist]

Study population
A total of 247 youth and 189 caregivers were enrolled and randomized.

Data management
Trial data were downloaded from REDCap on September 12, 2021. A separate dataset was generated for separately each study outcome in a "wide" format with the following structure. One row is generated for each of the 247 youth-caregiver pairs. Each variable is listed in a separate column, starting with youth responses at T1, T2, and T3, followed by caregiver responses at T1, T2, and T3 (where applicable). Datasets were exported in three formats: CSV, SAS, and SPSS.

A separate set of SAS datasets will be generated and sent to ICES. All string variables will be either recoded or removed.

Statistical methods
Statistical analyses will be performed using SAS Enterprise Guide version 7.1 (SAS Institute Inc., Cary, NC, USA) and R version 3.6.3. Descriptive statistics will be used to summarize participant (child and caregiver) characteristics at baseline, overall and by treatment arm. Frequencies and

proportions will summarize categorical measures. Medians and ranges will summarize continuous measures.
Mean imputation will be used to impute missing items from outcome scales. For the primary outcome (CIS), missing or “N/A” item-level responses will be considered as missing. The CIS score will be calculated as:

$$\text{CIS total score} = [\Sigma(\text{non-missing items}) / (\text{number of non-missing items})] * 13$$

A similar strategy will be used for the other outcome scales.

Statistical analyses will adhere to the intent-to-treat principle. To address the primary objectives, separately in youth and family members, the primary endpoint (CIS) will be compared between treatment arms across three time points using linear mixed models with random intercepts with an unstructured covariance matrix. The primary model will include: treatment arm, time (as a categorical measure), and treatment arm*time interaction. The primary model will be unadjusted. For the primary outcome, two-sided p-values < 0.05 will be considered statistically significant.
As sensitivity analyses, we will also perform the following models:

- Primary model adjusting for the following covariates: sex of youth (male/boy vs. female/girl vs. not male or female), age of youth at T1, and enrolling study site

Each secondary outcome will be analyzed similarly. Non-normally distributed outcome measures will be log-transformed. If the outcome distribution remains non-normally distributed after log-transformation, the outcome measure will be dichotomized at a pre-defined cutpoint and analyzed using generalized estimating equations (GEE). Two-sample t-tests were used for between-arm comparisons at a single timepoint.

For the secondary outcomes, two-sided p-values < 0.05 will be considered statistically significant. No adjustment will be performed for multiple hypothesis testing; findings will need to be validated in an independent study.

To identify heterogeneous treatment effects (HTE), please see the separate YouthCan Subgroup Analysis Plan (not yet performed).
