# Supplementary material for: Integrated Collaborative Care for Youths With Mental Health and Substance Use Challenges: A Randomized Clinical Trial
Source: JAMA Netw Open. 2025 May 13;8(5):e259565. doi: 10.1001/jamanetworkopen.2025.9565 (PMC12076176; doi:10.1001/jamanetworkopen.2025.9565)
Supplement: Supplement 3. — Data Sharing Statement [file jamanetwopen-e259565-s003.pdf]

Data Sharing Statement

Henderson. Integrated Collaborative Care for Youths With Mental Health and Substance Use Challenges. *JAMA Netw Open*. Published May 13, 2025.
doi:10.1001/jamanetworkopen.2025.9565

Data
Additional Information: ClinicalTrials.gov, NCT02836080,
https://clinicaltrials.gov/study/NCT02836080
Data available: Yes
Data types: Data dictionary
How to access data: Data dictionary will be shared upon reasonable request to the corresponding author
When available: With publication

Supporting Documents
Document types: None

Additional Information
Who can access the data: Researchers whose proposed use of the data has been approved, with permission obtained from each participating study site.
Types of analyses: The types of analyses will be made available for a specified purpose.
Mechanisms of data availability: Permission must be obtained from each participating study site to access study data.
